# Language Experience Predicts Eye Movements During Online Auditory Comprehension

**DOI:** 10.5334/joc.285

**Published:** 2023-06-28

**Authors:** Ariel N. James, Colleen J. Minnihan, Duane G. Watson

**Affiliations:** 1 Department of Psychology, Macalester College, US; 2 Department of Psychology and Human Development, Peabody College, Vanderbilt University, US

**Keywords:** Eye movements, Sentence processing, Reading, Working memory, Cognitive Control, Auditory word processing

## Abstract

Experience-based theories of language processing suggest that listeners use the properties of their previous linguistic input to constrain comprehension in real time (e.g. MacDonald & Christiansen, 2002; Smith & Levy, 2013; Stanovich & West, 1989; Mishra, Pandey, Singh, & Huettig, 2012). This project investigates the prediction that individual differences in experience will predict differences in sentence comprehension. Participants completed a visual world eye-tracking task following Altmann and Kamide (1999) which manipulates whether the verb licenses the anticipation of a specific referent in the scene (e.g. The boy will eat/move the cake). Within this paradigm, we ask (1) are there reliable individual differences in language-mediated eye movements during this task? If so, (2) do individual differences in language experience correlate with these differences, and (3) can this relationship be explained by other, more general cognitive abilities? Study 1 finds evidence that language experience predicts an overall facilitation in fixating the target, and Study 2 replicates this effect and finds that it remains when controlling for working memory, inhibitory control, phonological ability, and perceptual speed.

Spoken language is processed rapidly and incrementally during comprehension. Some of the evidence for this comes from the visual world paradigm (VWP), where eye movements to a visual display are measured as speech unfolds (Cooper, 1974; Tanenhaus, Spivey-Knowlton, Eberhard, & Sedivy, 1995). Typically, listeners make eye movements toward objects in the visual display as soon as there is sufficient linguistic information to identify the referent. Listeners can use a variety of cues to anticipate which object will be named, including word onsets (e.g. Allopenna, Magnuson, & Tanenhaus, 1998), predicates such as adjectives and verbs (e.g. Sedivy, Tanenhaus, Chambers, & Carlson, 1999; Altmann & Kamide, 1999), and converging cues from sentential context (e.g. Kamide, Altmann, & Haywood, 2003; Borovsky, Elman, & Fernald, 2012). While it is clear that language comprehension is incremental, questions remain as to which mechanisms support rapid processing. Previous work suggests a link between language experience (operationalized as, e.g., receptive vocabulary, productive vocabulary, or formal literacy attainment) and the ability to anticipate upcoming linguistic material. Much of this work measures individual differences among children developing literacy (Fernald, Perfors, & Marchman, 2006; Borovsky et al., 2012; Mani & Huettig, 2012; 2014) or compares adults of high and low literacy attainment (Huettig, Singh, & Mishra, 2011; Mishra, Singh, Pandey, & Huettig, 2012), although recent work has also measured individual differences among literate adults (Rommers, Meyer, & Huettig, 2015; Hintz, Meyer, & Huettig, 2017). The goal of the current work was to replicate the experience-anticipation link in literate adults, and to investigate what this link reveals about the mechanisms underlying efficient language processing. Importantly, our design employed the measurement of multiple constructs with multiple tasks to assess each one; this allowed us to test multiple proposed mechanisms simultaneously and robustly.

In this paper, we present two studies that tested (1) whether there are reliable individual differences in eye movement patterns among literate adults in a VWP following Altmann & Kamide (1999) Experiment 1; (2) whether these differences are related to variability in language experience; and (3) whether any link between eye movements and experience is explained by domain-general abilities such as processing speed or executive control. First, we present a brief overview of literature reporting a link between efficient language-mediated eye movements and language experience.

## Children

Work with young children has shown that differences in vocabulary predict anticipatory looks during language processing. Fernald and colleagues (1998) found that by two years of age, infants are able to look toward named targets before the entire word is completed, demonstrating rapid phonological processing. In a separate longitudinal study, Fernald and colleagues (2006) found that young children who had more rapid productive vocabulary growth during their second year tended to be faster and more accurate at identifying spoken targets in the looking-while-listening task at 25 months. The authors propose a link between processing efficiency and vocabulary knowledge although the direction of causality is unclear, i.e. whether a large vocabulary makes language processing more efficient or whether children who process language more efficiently tend to have larger vocabularies.

A 2012 study from Borovsky, Elman, and Fernald sheds some light on the nature of the experience-efficiency link. Borovsky and colleagues presented 3- to 10-year-old children and adults with a task in which the combination of semantic information about the agent and the verb uniquely identified a target object in a four-quadrant visual world display. For example, one scene pictured bones, a treasure chest, a ship and a cat. The agent PIRATE is semantically related to both the ship and the treasure, and the verb HIDE most likely refers to the bones or the treasure, and so the full context “The pirate hides the…” licenses anticipation of “treasure” only. Borovsky and colleagues found that receptive vocabulary size, not participant age or sentence completion scores, predicted anticipatory looking in this task. Further, it is critical to note that performance was related to vocabulary *relative to one’s age* and not raw vocabulary scores. This finding is counter to the idea that knowing more words leads to faster processing in the task. If that were the case, knowing more words should predict more anticipation, regardless of age. Instead, the authors argue, this finding suggests that faster processing contributes to both faster vocabulary learning and more anticipation during online sentence comprehension.

In contrast, Mani and Huettig (2014) focused on the role of language-specific mechanisms that aid efficient online comprehension. They found that the ability to read aloud real words, but not pseudowords, predicted 8-year-olds’ anticipation in a visual world task. Mani and Huettig argued that literacy provides orthographic representations for spoken words that are already known, and that this additional source of information boosts word recognition. Faster word recognition then allows resources to be allocated toward anticipating upcoming input. These results complement an earlier study by the authors, in which productive vocabulary predicted anticipatory eye movements (Mani & Huettig, 2012).

Overall, in work on children’s anticipation reviewed above, there is evidence that vocabulary size and anticipation are linked, and there are different proposed explanations for this link. On the one hand, domain-general processing speed may aid in both vocabulary development and rapid, anticipatory online comprehension (Borovsky, Elman, & Fernald, 2012). On the other hand, learning to produce (Mani & Huettig, 2012) and read (Mani & Huettig, 2014) words may deepen lexical representations, aiding in efficient online processing. Importantly, these explanations are not mutually exclusive.

## Adults with low literacy

Work from Falk Huettig and colleagues has demonstrated online processing differences between adults with higher and lower literacy attainment. Huettig, Singh, and Mishra (2011) compared (literate) undergraduate students to adults with low literacy, all of whom were native speakers of Hindi (the study took place in India). In two experiments, participants completed a look-and-listen task in which a target object was named at the end of a carrier phrase and four objects were displayed on screen. The question of interest was whether literacy impacts the kinds of competitors one considers during comprehension. As such, in both experiments, critical trials did not contain the target object. Displays in Experiment 1 contained a cohort competitor (shared phonological onset), a semantic competitor, and two unrelated distractors. While both high and low literates increased fixations toward the semantic competitor over the course of each trial (although high literates’ preference was of higher magnitude), only the high literates showed an early preference for cohort competitors, with low literates showing no significant difference between cohort competitors and unrelated distractors over the course of the trial. Semantic competitors were eliminated in Experiment 2; displays contained a cohort and three unrelated distractors. Again, high literates showed an early preference for cohort competitors that was time-locked to the unfolding speech; unlike in Experiment 1, low literates showed a marginal preference for cohort competitors over distractors, but it was small and delayed. The authors concluded that literacy refines phonological representations, allowing for the rapid, efficient use of phonological information during processing.

A later study by Smith, Monaghan, and Huettig (2014) used a computational model to test this phonological refinement account against a more general cognitive efficiency account. Consistent with Huettig and colleagues’ (2011) interpretation, manipulating the granularity of phonological representations in the model matched the literacy effects on phonological competitors, while the manipulation of cognitive efficiency did not have an impact on phonological processing.

There is also evidence that adults with low literacy make less effective use of other sources of information. Mishra, Singh, Pandey, and Huettig (2012) provided cues that uniquely identified a target object among three distractors. Sentences were spoken in Hindi; the structure of each sentence had the form “Right now you are going to” + ADJECTIVE + PARTICLE + TARGET NOUN + “see”, or roughly, “You will now see a(n) ADJECTIVE + TARGET NOUN”. Importantly, the target object was the best semantic match for the adjective, and was the only gender match for the gender-marked adjective and particle. Therefore, there was both semantic and grammatical information available to uniquely identify the target object before it is was named. The authors found that while highly literate adults used this constraining information to make anticipatory looks to the target, adults with low literacy attainment did not preferentially look at the target until it was named. These results, taken together with those earlier in this section, suggest that formal literacy enhances phonological and lexical representations that in turn enable rapid use of linguistic cues during auditory language processing.

## Adults with high literacy

The findings described so far suggest that those with low or developing literacy show less evidence of anticipatory processing than their more linguistically skilled peers. A question that arises is whether this relationship between language experience and anticipatory eye movements would still hold within a population of literate adults. Put another way, once a person reaches typical adult-like literacy and proficient language use, do individual differences beyond this point still predict anticipation? This is a theoretically important question for at least two reasons. First, these results could clarify whether anticipation can be thought of as a skill that can continue to improve along with increased language experience, rather than an ability that one is either proficient in or not. Second, focusing on literate adults eliminates the issues inherent in comparing adults of high and low literacy attainment; namely, adults with low literacy are likely to differ from adults with high literacy in a number of ways outside of literacy attainment *per se* (e.g. familiarity with being in a lab setting, socio-economic status).

A few studies from Huettig and his colleagues have investigated differences in anticipation within literate Dutch-speaking adults. Rommers, Meyer, and Huettig (2015) investigated individual differences in anticipatory eye movements among literate adults. Eighty-one adult participants completed a look-and-listen VWP task in which spoken sentences ended in a predictable target word. Scenes varied across three within-subject conditions; all scenes contained three unrelated distractor objects and either (1) the target, (2) a shape competitor, or (3) an additional unrelated control object. The researchers were interested in predictors of individual differences in anticipatory eye movements to both targets and shape-related distractors, the latter of which isolates the pre-activation of general visual forms from other features of the target. They found that linguistic predictors (higher receptive vocabulary and higher verbal fluency) were related to more target fixations, while a measure of non-linguistic anticipatory attention (the Posner Cueing task; Posner et al., 1978) was related to more shape-competitor fixations. The authors concluded that there are likely multiple mechanisms underlying language-mediated eye movements, and that these are differentially related to linguistic and non-linguistic factors.

Huettig and Janse (2016) focused on the roles of memory and processing speed in predicting anticipatory eye movements. Participants completed two verbal short-term memory tasks (nonword repetition and backwards digit span); one spatial short-term memory task (Corsi blocks; Corsi, 1972); two processing speed tasks: digit-symbol substitution (Wechsler Adult Intelligence Test, 2004) and letter comparison (Salthouse, 1996); and a *g-*loaded non-verbal intelligence measure (Raven’s Progressive Matrices; Raven, Raven, & Court, 1998). The researchers asked whether performance on these tasks was related to participants’ tendency to anticipate target words given cues to grammatical gender (the article *de* or *het*). They found that higher short-term memory and faster processing speed independently predicted more anticipatory looks, even when intelligence scores were entered into the model. Huettig and Janse concluded that models of predictive language processing must take memory and processing speed abilities into account.

Finally, Hintz, Meyer, and Huettig (2017) also found support for a multiple-mechanism account for language-mediated eye movements, although their focus was on item properties as well as individual differences among participants. In three experiments, participants completed a look-and-listen VWP task in which prediction of the sentence-final target object was facilitated or not (in the style of Altmann & Kamide, 1999). Objects were presented in a four-quadrant display including the target and three unrelated distractors. Across the three experiments, receptive vocabulary was a robust predictor of anticipatory eye movements. Verbal fluency was only a predictor when participants had one second of preview prior to the occurrence of the verb in the spoken sentence (Experiments 1 and 2). Performance on Raven’s Progressive Matrices was not a robust predictor of eye movements when the verbal measures were included in the model.

Across the three articles just reviewed, there is consistent evidence that there are predictable individual differences in anticipatory language-mediated eye movements, even among literate adults. Some of the factors that predict this variability align with those implicated in the studies of children and adults with low literacy: receptive vocabulary (Rommers et al., 2015; Hintz et al., 2017) and perceptual speed (Huettig & Janse, 2016). There is also support for a role for short-term memory (Huettig & Janse, 2016), general attentional control (Rommers et al., 2015), and verbal fluency, although it’s unclear whether the last factor is only relevant when participants have longer (one-second) previews, as in Hintz and colleagues’ (2017) (but see the Rommers et al., 2015 positive verbal fluency result with a 500-ms preview). Finally, across two studies, performance on Raven’s Progressive Matrices failed to explain variability in eye movements when other factors were included in the models (Rommers et al., 2015; Hintz et al., 2017). One difficulty in summarizing these findings is that the visual world tasks differ in terms of the timing and type of anticipatory cues; another is that the constructs with positive findings have not all been collected in the same study, which would clarify whether they explain unique variance in a particular language processing effect. The current set of studies will begin to address these points.

## The current study

### Question 1

The current study investigates individual differences in anticipatory language-mediated eye movements among literate adults. Taking a step back from predicting individual differences, our first question is whether there *are* reliable individual differences. Although the work reviewed in the previous section suggests that there are individual differences (Rommers et al., 2015; Hintz et al., 2017), the by-subject reliability of the dependent measure in the VWP has not been explicitly demonstrated. Put another way, we do not know the extent to which an individual’s eye movements in a VWP task represent something stable about their ability. This question is important because the reliability of the eye movement measure sets an upper bound for the correlations we can expect from the predictors of interest.

Replicability of an experimental effect (e.g. the robust prediction effect from Altmann & Kamide, 1999 and later replications) does not guarantee reliability at the individual participant level. This issue was highlighted in James, Fraundorf, Lee, and Watson (2018), who described this issue in self-paced reading times. In fact, more robust experimental effects may be *less* likely to produce stable individual differences, an issue referred to by Hedge, Powell, and Sumner (2018) as the “reliability paradox”. As the authors explain, experiments and correlational studies have conflicting meanings of “reliability” – a reliable experiment is one in which participants tend to show an effect to a similar degree, while reliability in correlational studies depends on the ability to consistently *rank* individuals (p. 1167). Hence, robust experimental effects rely in part on *low* between-subject variance, while robust correlational effects depend in part on *high* between-subject variance. This conflict between the experimental and correlational approaches was captured in Lee Cronbach’s 1957 description of the “two-disciplines problem”.

Thus, we acknowledge that the reliability of our VWP measure for the purposes of assessing individual differences is an empirical question, and we investigate this explicitly. Farris-Trimble & McMurray (2013) investigated test-retest reliability in looks to target objects and different types of phonological competitors in a four-quadrant visual world task. They estimated growth curves in proportions of fixations for each individual subject and object type. They found moderate reliability overall in the time course of eye movements, but this varied as a function of the type of object (target, cohort, or rhyme) and the parameter of the time course curve (e.g. timing and height of peak proportion of fixations). While these data are promising for the current investigation, they do not tell us the reliability of *effects* within participants. That is to say, is a participant’s *difference* in the eye movement record between targets and competitors consistent? While a formal test of reliability (e.g. a test-retest design) was outside of the scope of the current project, we do investigate internal consistency and make recommendations for future investigations. There is not a widely-accepted standard practice for computing the internal consistency of cognitive experimental effects (see Pronk, Molenaar, Wiers, & Murre, 2022 for discussion), so we present two different, complementary approaches. First, we asked whether models predicting our eye movement outcomes were improved by including random by-subject slopes for condition; if participants reliably differ from one another in the size of their condition effects, random slopes will improve the models’ ability to explain variance in the outcome. Second, we asked whether each participant’s condition effect was consistent across split halves of the critical items; following Pronk et al. (2022), we created random split halves of the critical trials, balanced by condition, and computed the average correlation between the effect sizes obtained from each half. Further details of both procedures are described in the Results sections of both studies.

### Question 2

Our next question is whether we can replicate the link between language experience and anticipatory eye movements that is suggested by the studies reviewed above. Similar to Rommers et al. (2015) and Hintz et al. (2017), we focus our investigation on literate young adults in a simple VWP task. As in Hintz et al., we measure anticipation by following Altmann & Kamide (1999), presenting subjects with displays containing a single target among unrelated distractors. On half of the critical trials, anticipation of the target is licensed by semantic information in the verb. As in Altmann & Kamide Experiment 1 and unlike Hintz et al., participants were asked to select at the end of every trial whether the target was present, rather than look and listen.

Language experience was defined broadly in the current study as a combination of both skills (e.g. vocabulary knowledge) and experience (e.g. time spent reading). We chose tasks that were designed to probe differences between individuals that could arise from various linguistic sources (i.e. vocabulary knowledge can be gained from reading, but also listening), although we attempted to bias the battery toward experience with texts. We made this choice both (a) to be able to use measures in common with previous work outlined above and (b) because we felt that it would be more theoretically compelling to demonstrate that behavior in a listening task with simple words and sentence structures could be related to language experience that is biased toward a different modality. Further details about each of our five language measures are provided in the Study 1 Method section.

### Question 3

Finally, our third question is whether any relationship between language experience and anticipatory eye movements can be explained by other abilities, including speed of processing (raised in the discussion of children and literate adults) and phonological abilities (raised in the discussion of adults with low literacy), as well as inhibitory control (Rommers et al., 2015) and working memory (Huettig & Janse, 2016). We address this set of questions in Study 2.

## Study 1

**Figure 1:**
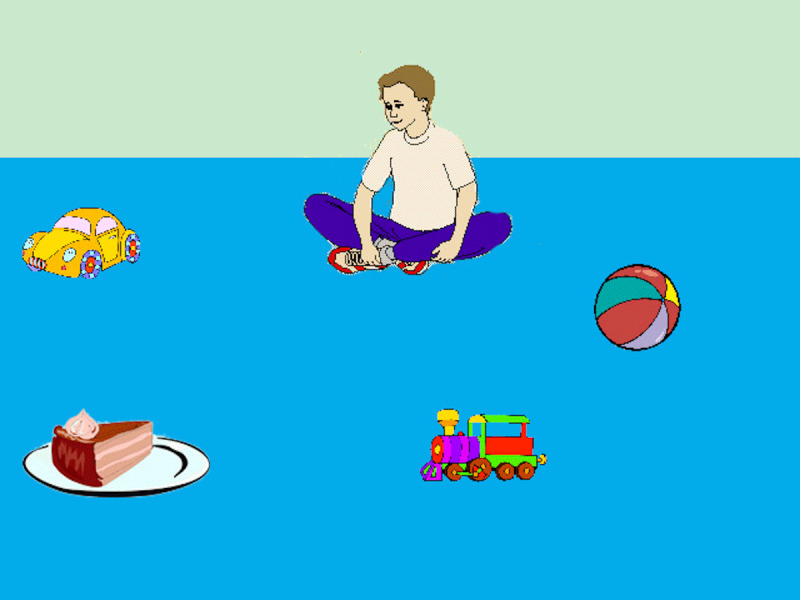
Display for the sentence *The boy will eat the cake* or *The boy will move the cake*. *Note*: Images were updated from the original Altmann & Kamide (1999) stimuli.

The first study investigated the relation between language experience and spoken language processing in a visual world task in which the target object could be selected based upon the semantics of the preceding verb. In a replication of the Altmann and Kamide (1999) paradigm, participants viewed scenes such as Figure 1 and heard either a constraining sentence such as (1) or a less constraining sentence (2). At the verb in (1), a listener is able to anticipate that *cake* is a likely object, as it is the only edible object in the scene. In contrast, the verb in (2) does not license anticipation of a specific object, as all of the objects in the scene can be moved by the boy.

1. The boy will *eat* the cake.
2. The boy will *move* the cake.

If language experience within literates is related to efficient spoken processing, the measures of linguistic experience should track fixations in this simple task. Additionally, the two trial conditions allowed us to look both at the effect of semantic constraint at the verb (*eat* versus *move*), and main effects of language experience on fixations (e.g. latency to fixate the target across conditions).

### Method

#### Participants

Participants were undergraduate students at the University of Illinois in Urbana-Champaign participating for class credit. They were all native speakers of English with normal hearing and normal or corrected-to-normal vision. One hundred and twenty-four subjects participated in the study; 13 were dropped due to missing data (three were missing one of the language experience measures due to computer failure, 10 were missing all eye-tracking data due to calibration failure), resulting in 111 participants included in the analyses. One person did not report demographic information. Of the remaining 110 participants, 70 self-identified as female, 40 as male, and the average age was 19 years and 2 months (range: 18–22 years).

#### Materials

Measures for the language experience assessment, and stimuli for the VWP task are described below.

##### Language experience

Language experience was measured with five different tasks. The primary goal in selecting these five particular tasks was to find a diverse set of measures that do not specifically probe sentence comprehension or prediction, but instead capture other aspects of behavior that we expected to vary according to individuals’ experience.

##### Author Recognition Test

The Author Recognition Test (ART) was developed as a measure of exposure to print materials (Stanovich & West, 1989). In the current study, we used an updated and slightly lengthened version of the task developed by Acheson, Wells, and MacDonald (2008) that included 65 authors’ names and 65 foil names. In their version, all 130 names were randomized and presented to participants on a sheet of paper and participants circled the names that they believed belonged to the authors of books. For the current study, the test was adapted for the computer. Participants in the current study saw names presented one at a time and made a judgment about each name. Names were presented in a random order, and two response buttons appeared at the bottom of the screen reading “Author” and “Don’t know”. Participants were told that there was a penalty for guessing, so they were encouraged to only respond with “Author” if they were sure, and to otherwise choose “Don’t know”.

##### Extended Range Vocabulary Test

The Extended Range Vocabulary Test (ERVT; Ekstrom, French, Harman, & Dermen, 1976) includes 48 words of varying difficulty. Participants chose which among five single words has the most similar meaning to the given word. Participants were told that there would be a penalty for guessing incorrectly, so they were encouraged to select a sixth “Not sure” option if they were unfamiliar with the word.

##### North American Adult Reading Test

The National Adult Reading Test was developed as a way to estimate pre-morbid IQ in brain trauma patients (Nelson, 1982) and adapted for North American participants by Blair and Spreen, (1989). We used the latter North American Adults Reading Test (NAART) in the current study. Participants received a list of 61 words with irregular spellings, presented one at a time at increasing difficulty. The participants’ task was to read the word and correctly pronounce it. Participants’ responses were scored as correct if they matched one of the pronunciations provided in the Merriam-Webster online dictionary (Merriam-Webster.com, 2012), and as incorrect otherwise; no partial credit was given. If participants produced more than one response for a given item, only the last attempt was scored. Success in this task depends on participants’ familiarity with both the written form of the word and the accepted pronunciation and thus cannot be neatly categorized as a test of print exposure (participants may have read and understood a word before but not know the pronunciation) nor as a test of listening exposure (participants may have used and understood a word but be unfamiliar with its written form). In spite of the test’s unique nature, scores on the NAART have been shown to relate to more widely-used measures of verbal ability (Uttl, 2002; Blair & Spreen, 1989).

##### Comparative Reading Habits

Comparative Reading Habits (CRH) is a survey in which participants answer five questions comparing their own reading habits to what they perceive to be the norm for their fellow college students (Acheson et al., 2008).

##### Reading Time Estimate

Reading Time Estimate (RTE) is a survey in which participants estimate how many hours in a typical week they read various types of materials, including fiction, newspapers, and online materials (Acheson et al., 2008).

##### Eye-tracking

The design of the eye-tracking task closely followed that of Altmann and Kamide (1999). Sixteen scenes were created using Photoshop and cartoon images from the ClipArt database. Two sentences were recorded for each of these scenes, one with a predictive verb and one with a neutral verb. For instance, for a scene with a boy sitting on the floor surrounded by a toy train, toy car, ball, and a piece of cake (see Figure 1), participants heard either *The boy will eat the cake* or *The boy will move the cake*. Scenes either contained four or five total objects. In scenes with five objects, one object did not make sense in either sentence context. An additional 16 filler scenes were created such that the target object described in the sentence was not present in the scene. We thank Altmann and Kamide for making the original sentences and scenes available. The 16 critical sentences were taken from Altmann and Kamide (1999) and the corresponding scenes were edited and re-colorized for the current study. The 16 filler scenes and sentences were created for the study. All sentences were recorded by the same female speaker of Midwestern American English and read at a natural speech rate. Durations for critical words are given in Table 1. A full list of the Study 1 stimuli are presented in Appendix A.

**Table 1:** Mean word durations (rounded to nearest ms) for sentence stimuli in Studies 1 and 2, along with the values from Altmann & Kamide (1999, Table 2, p. 254) for comparison.


	PREDICTIVE CONDITION				NEUTRAL CONDITION			
	
	VERB	BREAK	“THE”	TOTAL	VERB	BREAK	“THE”	TOTAL

Study 1	523	*–*	147	670	551	*–*	137	688

Study 2	489	*–*	155	644	499	*–*	154	653

A & K	383	192	122	697	423	180	107	710


*Note*: Sentences in Altmann & Kamide (1999) included a post-verb break; the current studies did not.

#### Procedure

All participants completed the tasks in the same order to minimize variability between subjects that is due to differences in the experimental session (see Swets, Desmet, Hambrick, & Ferreira, 2007 for discussion). Participants completed the ART, the ERVT, the NAART, the CRH, the RTE, and then the eye-tracking task. The entire procedure took 35 to 50 minutes.

##### Language experience

All language experience measures were programmed and displayed using the Matlab Psychophysics Toolbox (Brainard, 1997), on the same computer as the eye-tracking task. All of the language experience measures together typically took 15 to 30 minutes for participants to complete.

##### Eye-tracking

Participants were seated at an Eyelink 1000 desk-mounted eye-tracker. Their heads were stabilized using a chin rest. Participants were instructed to decide whether the recorded sentence could be a possible description of the scene. They were instructed that they should respond *yes* to *The man will choose the watch* if there was a watch in the corresponding scene, and *no* otherwise. Before calibration, participants completed a practice trial in which they viewed a scene and heard the sentence *The man will light the candle*. After the participants chose a response, they were told that they should have responded *yes* because there was a candle present in the scene, even though there was no visible lighter or match.

Participants then completed a calibration procedure and began the task. Before each trial, the eye-tracker was recalibrated by having the participant fixate a centrally presented white dot on a black screen.

### Results

**Figure 2:**
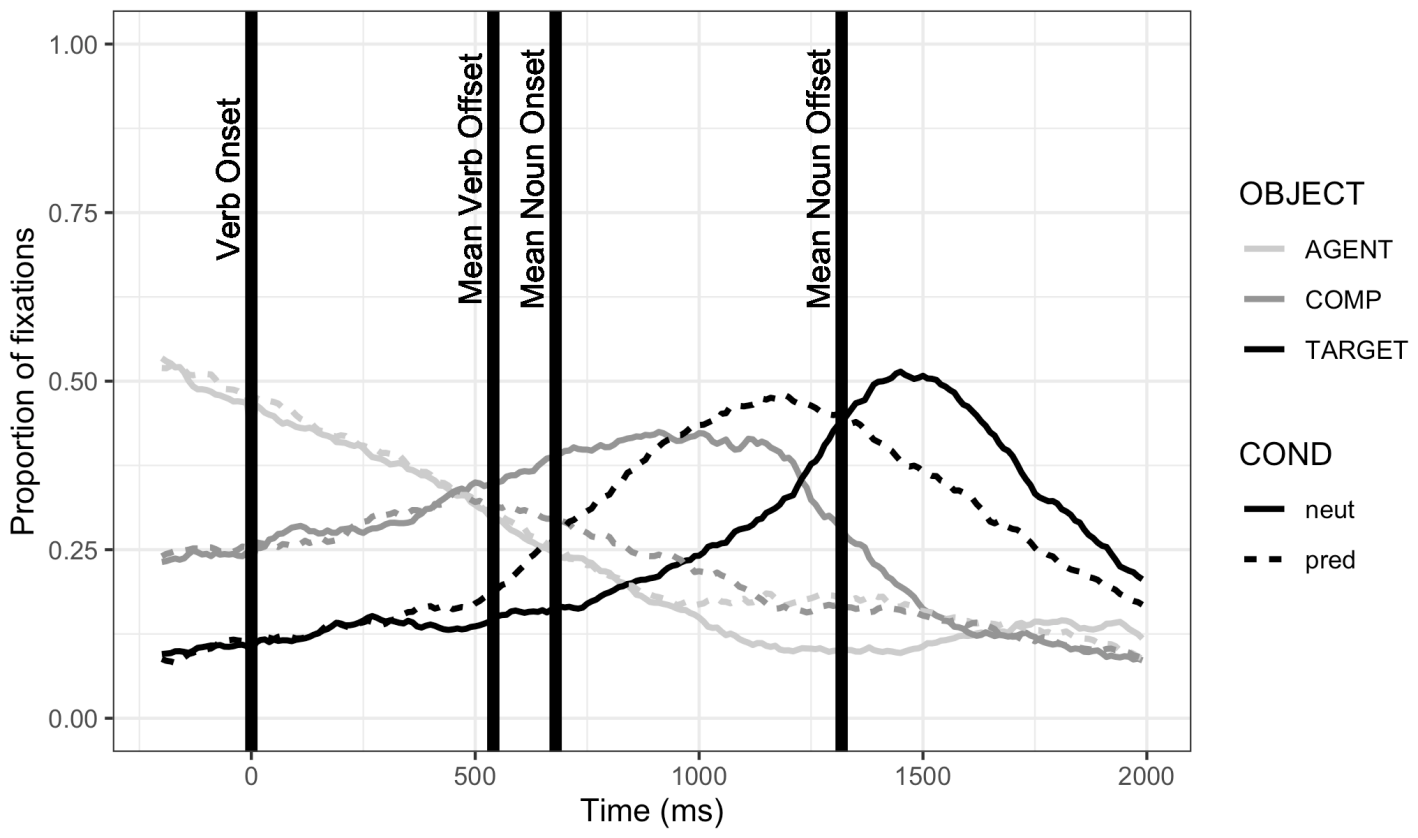
Study 1: Proportion of fixation durations to regions of interest, by condition. *Note*: The y-axis presents the proportion of each 10-ms bin that was spent fixating the regions of interest (ROIs): the agent (e.g. the boy), the target (e.g. the cake), and any of the competitor objects (e.g. the sum of fixations to the car, ball, and train). Nonsense objects, which were included in half of critical trials, were included in the total of competitor fixations. The total proportion of fixations within a bin does not sum to one because of the time spent looking outside of the ROIs. The x-axis presents time starting from 200 ms before the verb onset, which is aligned at 0 ms; the means of verb offset and noun on- and offset times are shown for illustrative purposes.

The eye-tracker failed to record 26 trials (0.74%). Regions of interest (ROIs) were defined by drawing a close-fitting rectangle around each object, including the agent (e.g. the boy in Figure 1); participants’ fixation coordinates were then categorized by object such that any fixations falling outside of any ROIs were not counted. Figure 2 plots fixations to these ROIs over time.

#### Latency

**Figure 3:**
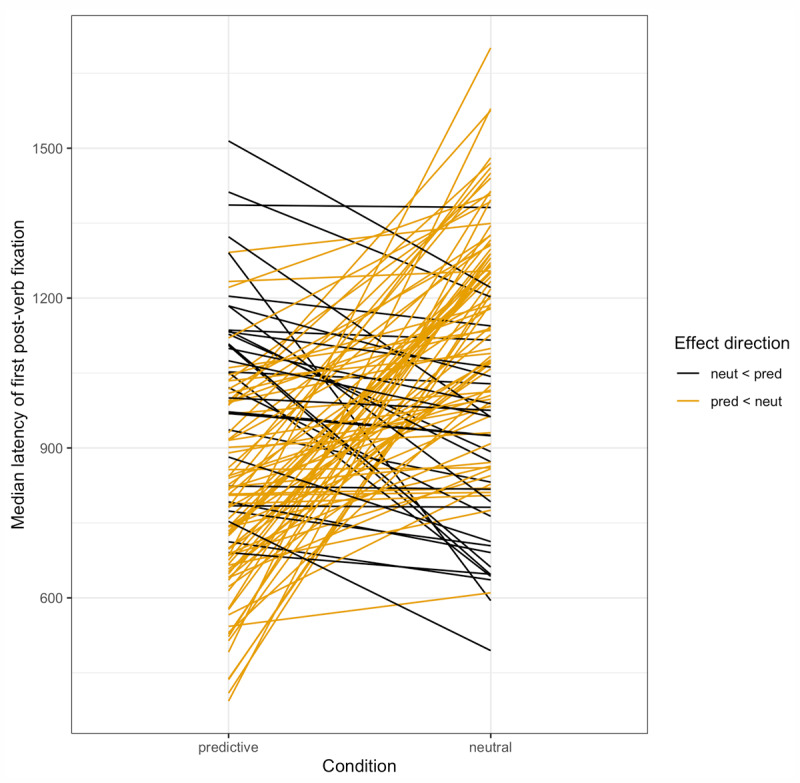
Study 1: Median latency to fixate the target by condition by subject.

To test whether we replicated the basic anticipation effect from Altmann & Kamide (1999), we asked how soon after the verb onset did participants first fixate the target object, and whether this latency varied by verb condition (predictive, e.g. *…eat the cake* vs. neutral, e.g. *…move the cake*). For all latency analyses, we were interested in fixations that could have been initiated as a result of comprehending the verb. For this reason, we only included fixations that began after the verb onset *plus 200 ms* to account for the average ocular-motor delay (Viviani, 1990). Across all trials, there were 500 in which at least one target fixation started and ended before the cut-off time (31.5% of predictive trials, 33.2% of neutral trials); such trials were retained, given that there was a later fixation that started after the cut-off time that would then be used for the latency analyses. Across all trials, there were 229 in which a target fixation was initiated before this cut-off time and persisted into the verb time window (predictive trials 14.3%; neutral trials: 15.4%); these were excluded from analysis. Of the remaining trials, there were 137 in which the target ROI was not fixated at all after the verb (predictive trials 7.3%; neutral trials: 10.5%); of these 137 trials, there were 17 in which the target had been fixated earlier in the trial (predictive trials: 12.3%; neutral trials: 12.5%). A total of 1316 latencies were included in the following regression analyses. The mean latencies by condition are given in Figure 3.

#### Fixation probability

**Figure 4:**
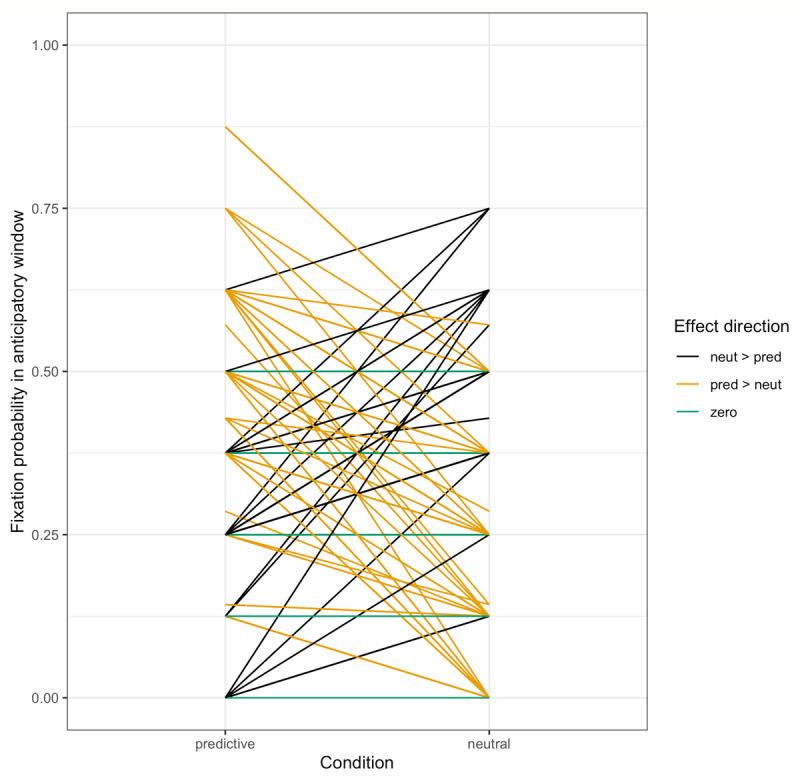
Study 1: Target fixation probability by condition by subject.

Following Altmann & Kamide (1999), we also investigated fixations that occurred before the onset of the noun. While the latency analyses test the prediction that target fixations will be faster following a verb that licenses anticipation, they include fixations that occurred after the target noun was said. In contrast, the proportion-of-fixations analyses specifically ask whether participants are more likely to fixate the target *before* they hear the name of the target. We defined the beginning of the anticipatory window as the verb onset plus the 200-ms ocular-motor delay, and the end as the noun onset (without the 200-ms delay, to be conservative). We calculated a proportion-of-fixations measure by taking the total duration of any target fixations in the anticipatory window and dividing it by the total duration of fixations to all objects, including the agent. Because the proportion of target fixations in the anticipatory window was 0 on a large number of trials (1031 of 1680, 65.2%, which includes 77 trials in which there were no fixations registered in any of the ROIs), we chose to convert these proportions into a binary measure: 1 if the target was fixated at all in this window and 0 if not, generating what will be referred to hereafter as the *fixation probability*. Counts of trials with and without target fixations, broken down by condition, are given in Table 2. The average fixation probability in each condition by subjects is given in Figure 4.

**Table 2:** Trials With and Without Target Fixations in the Anticipatory Window.


		TARGET FIXATED	TARGET NOT FIXATED	NO LOOKS IN ROIS	TOTAL TRIALS

Study 1	Predictive	324 (39%)	489 (58%)	26 (3%)	839

	Neutral	261 (31%)	542 (64%)	38 (5%)	841

Study 2	Predictive	579 (32%)	1147 (64%)	68 (4%)	1794

	Neutral	0 (0%)	1379 (77%)	414 (23%)	1793


#### Language experience

Table 3 summarizes performance on the five different measures of language experience. Excluding the two survey measures, we calculated split-half correlation estimates of the language experience tasks to assess the internal consistency of the measures. Following Pronk and colleagues (2022) and using their splithalfr package, we derived the mean correlation of 1,000 randomly-generated split halves (see further details about this procedure below in our analyses for Question 1). This resulted in mean correlations of 0.59 (SD = 0.08) for ART, 0.68 (SD = 0.05) for ERVT, and 0.75 (SD = 0.03) for NAART. Because each item on the survey measures were designed to assess a different aspect of reading experience, calculating a split-half correlation is not appropriate. Instead, we present correlations among survey items within the CRH (Table 4) and RTE (Table 5). Table 6 presents the correlations among the five measures. With the exception of the Reading Time Estimate (RTE), the measures are reliably correlated with one another. We created a composite score by centering and standardizing each score, and then taking the average of all five.

**Table 3:** Performance on Language Experience Tasks in Study 1.


MEASURE	POSSIBLE RANGE	OBSERVED RANGE	MEAN SCORE (SD)

ART	Min: –65	Min: 0	12.05 (6.34)

	Max: 65	Max: 30	

ERVT	Min: –12	Min: 1.5	13.97 (6.19)

	Max: 48	Max: 29.25	

NAART	Min: 0	Min: 10^a^	29.06 (7.68)

	Max: 61	Max: 48	

CRH	Min: 5	Min: 8	21.14 (4.32)

	Max:35	Max: 31	

RTE	Min: 0	Min: 6	19.77 (8.34)

	Max: 63	Max: 41	


*Note*: ART = Author Recognition Test, ERVT = Extended Range Vocabulary Test, NAART = North American Adult Reading Test, CRH = Comparative Reading Habits, RTE = Reading Time Estimate. ^a^ This subject accidentally skipped one item, so the score is out of 60. Proportion correct are used in the analyses.

**Table 4:** Correlations Among Comparative Reading Habits Items in Study 1.


	TIME	COMPLEX	ENJOY	UNDERSTAND

Complex	0.279**	–		

Enjoy	0.510***	0.214*	–	

Speed	0.196*	0.283**	0.424***	–

Understand	0.136	0.121	0.270**	0.498***


*Note*: Participants answered five questions in which they compared themselves with their peers on how much time they spend reading (“Tme”), how complex their reading material is (“Complex”), how much they enjoy reading (“Enjoy”), how fast they read (“speed”), and how well they understand the material when reading at their normal pace (“Understand”). + *p* < 0.1. * *p* < 0.05. ** *p* < 0.01. *** *p* < 0.001.

**Table 5:** Correlations Among Reading Time Estimate Items in Study 1.


	1	2	3	4	5	6	7	8

1. Textbooks	–							

2. Academic texts^a^	0.03	–						

3. Magazines	0.07	–0.02	–					

4. Newspapers	–0.05	0.28**	0.45***	–				

5. Emails	0.16	0.24*	0.22*	0.24*	–			

6. Websites^b^	0. 11	0.33***	0.17^+^	0.16^+^	0.49***	–		

7. Fiction	0.08	0.21*	0.18^+^	0.17^+^	0.07	0.09	–	

8. Non-fiction	0.02	0.24*	0.23*	0.30**	0.18^+^	0.06	0.59***	–

9. Other	0.09	0.31**	0.23*	0.34***	0.28**	0.21*	0.405***	0.49***


*Note*: Participants estimated how much time they spent reading different types of material in a typical week. + *p* < 0.1. * *p* < 0.05. ** *p* < 0.01. *** *p* < 0.001. ^a^ Other than textbooks. ^b^ Other than email.

**Table 6:** Correlations Among Language Experience Task Scores in Study 1.


	ART	NAART	ERVT	CRH

NAART	0.55***	–		

ERVT	0.66***	0.66***	–	

CRH	0.39***	0.34***	0.47***	–

RTE	0.17^+^	0.14	0.053	0.32***


*Notes*: ART = Author Recognition Test, NAART = North American Adult Reading Test, ERVT = Extended Range Vocabulary Test, CRH = Comparative Reading Habits, RTE = Reading Time Estimate. + *p* < 0.1. * *p* < 0.05. ** *p* < 0.01. *** *p* < 0.001.

#### Analyses

We pursued a multilevel mixed-effects regression approach for our analyses. We initially implemented this approach within a traditional frequentist framework, building our models with lme4 (Bates et al., 2015) and lmerTest (Kuznetsova et al., 2017). However, we pivoted to a Bayesian approach for two primary reasons. First, we wanted to be able to estimate the full random effects structure, in part for assessing the degree of individual differences in the outcomes, and we encountered convergence issues with our original analysis. In addition, we wanted to shift away from a focus on *p-*values; not only did we have concerns that the moderate reliability of our language experience scores could inflate Type I error (see Westfall & Yarkoni, 2016) but we also appreciated the shift in focus toward the evidence for estimated effects. The Bayesian approach estimates an entire distribution of values for each parameter, enabling the analyst to quantify how the data, in light of prior expectations, impacts the range of plausible values for the parameter (see Kruschke & Liddell, 2018 for a brief overview of applied Bayesian statistics). Our original frequentist analyses are presented in Appendix B and revealed qualitatively similar patterns of results.

All of the following analyses were completed using the brms package (Bürkner, 2017) in R Studio (version 2022.07.2). All of the regression models described below included the full random effects structure (by-subject and by-scene intercepts and slopes), unless otherwise stated. We specified weakly informative priors for all fixed effects, and the brms default priors for all other parameters. All simulations were run with two sampling chains for 8,000 or 10,000 iterations (1,000 warm-up iterations). For each model, we report the estimate for the parameter of interest; the range of parameter values that captures 95% of the posterior distribution for that parameter value (the 95% credible interval, or CI); and the proportion of estimated values that are greater than 0 (in the case of positive effects) or less than zero (in the case of negative effects).

#### Condition effect

To test whether latency to fixate the target was related to verb condition, we built a linear regression model of latency, log-transformed to approximate a normal distribution, predicted from verb condition (dummy coded with the neutral verb condition as 0) and random effects. This model resulted in an effect of condition such that the target was fixated sooner following the predictive verb (estimate = –0.19, 95%–CI = [–0.28; –0.10], *p*(<0) = 1.00).

To predict participants’ target fixation probabilities during the anticipatory window, we built a logistic regression model to predict the fixation probability from condition. The resulting model had a random effects structure that included only random intercepts for subjects and scenes. This model resulted in an effect of condition such that the target was more likely to be fixated following the predictive verb (estimate = 0.46, 95%-CI = [0.18; 0.73], *p*(>0) = 0.999).

#### Question 1: Reliability of individual differences in condition effects

After replicating the effect of verb condition across subjects, we turn to the first of our research questions: do we see evidence for stable individual differences in the size of the verb effect? Put another way, is there sufficient consistency *within* subjects to differentiate them from one another? We addressed this question in two ways: a model-based approach and a split-half approach. With our model-based approach, we asked whether having random slopes for subjects in our multilevel regression models is justified by the data. Specifically, we compared models with and without random slopes and computed a Bayes Factor (BF). For both latency and fixation probability, we built a full model with random slopes and intercepts for subjects (without estimating the correlation between the random effects), and a simpler model without the random slopes. For latency, the BF for random by-subject slopes was 0.037, meaning that the simpler model was preferred. This is evidence *against* the inclusion of random slopes. The same was true in predicting fixation probability (BF = 0.094). However, repeating this procedure for the random intercepts rather than slopes yielded support for random effects for both dependent measures (Latency: BF = 6.208; Probability: BF = 9.805). These results suggest that there is evidence for individual differences, but in overall latency or fixation probability rather than in the effect of verb condition. This should temper expectations that language experience will interact with verb condition. Still, we chose to retain the full random effects structure in our subsequent analyses, as it has been argued that this is beneficial for model estimation within both frequentist (Barr et al., 2013) and Bayesian (Oberauer, 2022) frameworks.

Our second approach to testing the reliability of individual differences in eye movements was to look at split halves of critical trials, following recommendations and the associated R package (splithalfr) described in Pronk et al. (2022). For each dependent measure, the general procedure was as follows: (1) each participant’s critical trials were split into halves, balanced by verb condition; (2) the difference between condition means was computed in each half; (3) the first two steps were repeated to create a total of 1000 pairs of difference scores per subject; (4) these pairs were used to estimate a Spearman-Brown-corrected Pearson correlation across subjects for each replication; and (5) we took the mean of these correlations as our estimate of internal consistency. For latency, the mean split-half correlation was 0.18 (SD = 0.12); for probability, the mean split-half correlation was 0.15 (SD = 0.12). In line with our model-based approach, internal consistency of the condition effect was low.

#### Question 2: Predicting eye movements from language experience

To address whether there is a link between anticipatory eye movements and language experience scores, we tested whether language experience scores interacted with the condition effects, both in predicting fixation latencies and fixation probability. The language composite score and its interaction with condition were added to both condition-effect models described above. In predicting both latency and fixation probability, the language-by-condition interaction was not supported by the data (Latency: estimate = 0.02, 95%–CI = [–0.05; 0.09], *p*(<0) = 0.302; Probability: estimate = 0.00, 95%-CI = [–0.34, 0.34], *p*(>0) = 0.503). However, for latency, there was some evidence for a main effect of language experience (estimate = –0.06, 95%-CI = [–0.12; 0.00], *p*(<0) = 0.978), such that higher composite scores were associated with faster target latencies across verb conditions. Evidence for a similar relation with fixation probability was considerably weaker, but not trivial (Probability: estimate = 0.20, 95%-CI = [–0.09, 0.49], *p*(>0) = 0.912).

### Discussion

Study 1 found that, across participants, fixations to target objects were faster when the verb semantics licensed anticipation; this is a replication of the critical effect in Altmann and Kamide (1999). For our current purposes, we asked whether there were individual differences in participants’ eye movements (Question 1). We found that, while overall latencies reliably varied across participants, there was not evidence for stable individual differences in the *condition* effect. Thus, we are not able to predict differences in anticipatory eye movements, but in overall speed. A composite score of language experience significantly related to those differences in speed (Question 2).

While it is possible that these results suggest that anticipatory eye movements, as indexed by the verb condition effect, do not vary meaningfully across individuals, another possibility is that we were unable to assess true underlying differences because our eye-movement measure lacked internal consistency. As an attempt to address this, Study 2 doubled the number of critical items to increase the precision of each individual’s estimated condition effect.

Why might language experience be related to fixation latencies generally? One possibility is that experience sharpens phonological representations of known words (Mani & Huettig, 2014; Smith et al., 2014), while another is that language experience is related to an overall boost in speed and cognitive efficiency (Smith et al., 2014; Borovsky et al., 2012). Still another possibility is that there is not an effect of language experience *per se*, and that language experience is merely correlated with an unmeasured factor that drives eye-movement patterns. Study 2 attempted to address this issue (Question 3) by including measures of phonological abilities and domain-general cognitive skills that could be related to both language experience and performance in the eye-tracking task.

## Study 2

The second study follows the same design as Study 1, but includes additional measures of individual differences to address the mechanisms underlying the experience-anticipation link found in Study 1. Measures of phonological processing were included, as previous work has highlighted phonological precision as a common thread tying together previous experience with spoken language, orthographic representations in print, and online word decoding. Working memory, inhibitory control, and perceptual speed, were included because they address a domain-general processing efficiency mechanism that may underlie the language experience effect found in Study 1. These factors have also individually been implicated in previous research on individual differences in sentence processing more broadly, lending support to the suggestion that these factors may play a role in online comprehension here.

The design of Study 2 allows us to simultaneously address the contributions of these different factors within individuals by including them all in the current study. A strength of this approach is that there are multiple measures of each of these five constructs, as no one measure is process-pure. If language experience *per se* guides eye movement behavior in spoken sentence processing, as is suggested by Study 1, the effect of experience should remain even after these other cognitive factors are accounted for.

### Method

#### Participants

Participants were adults from the University of Illinois in Urbana-Champaign community, participating for class credit or for $8 per hour. These participants also took part in the study described in James and colleagues (2018); thus, they also participated in a self-paced sentence-reading study that is unrelated to the current study. The majority (90% of those providing demographic information) were current undergraduates, and the remaining had at least a bachelor’s degree. Participants were all native speakers of English with normal hearing and normal or corrected-to-normal vision. One hundred and thirty-one participated in the study. A total of 31 subjects have missing data. Of those, 15 are missing eye-tracking data and are excluded from the analyses; seven failed calibration and eight did not show up for the second session of the study during which the eye-tracking task took place. The other 16 subjects are missing at least one individual differences measure; nine ran out of time during the session and were not able to finish the remaining tasks, five experienced a technical malfunction, and two misunderstood a task. Subjects that had at least one measure for each individual differences domain were included in the analyses; excluding these six subjects does not substantively change the results. Of the 106 participants included in the analyses, 75 self-identified as female, 31 as male, and the average age of these participants was 20 years and 10 months (range: 18–67; excluding the 67-year old participant, the average is 20 years and 5 months, and the maximum age is 35).

#### Materials

Measurements for the five different cognitive domains, and for the visual world eye-tracking task, are described next.

##### Language experience

Participants completed the same language experience battery as described in Study 1.

##### Phonological ability

In children and adults with low literacy, phonological processing skill has been proposed as a link between experience and online sentence processing efficiency (e.g. Fernald et al., 2006; Huettig, et al., 2011). In literate adults, phonological ability is a possible factor that underlies sentence processing skill, as the ability to create phonological representations may aid in the maintenance of the words that have been encountered so far, as is required in reading as well as verbal working memory tasks (MacDonald & Christiansen, 2002; Acheson & MacDonald, 2009, 2011). For this reason, we expect that phonological ability may be related to individual differences in language experience, as well as the working memory span tasks. It is also possible that the clarity of phonological representations may aid in the comprehension of the spoken sentences presented in the eye-tracking task, independent of differences in language experience. Phonological ability was assessed using three measures. Two of these, Blending Nonwords and Phoneme Reversal, are taken from the Comprehensive Test of Phonological Processing (CTOPP; Wagner, Torgesen, & Rashotte, 1999).

##### Blending nonwords

On each trial, participants heard a list of phonemes or syllables and were asked to combine these elements into one nonword. For instance, if the participants heard /h/, /ε/, and /t/, they would need to produce /hεt/ as one word. The number of elements ranged from two to eight. Participants were given six practice trials and 18 critical trials. Participants’ responses were scored as correct if they matched the pronunciation provided in the CTOPP manual, and incorrect otherwise. No partial credit was awarded.

##### Phoneme reversal

In the phoneme reversal task, participants heard nonsense words and were asked to repeat the word and then pronounce it backwards, creating a real English word. For instance, if the participants heard /stuːb/, they would need to produce the word “boots”. Participants were given four practice trials and 18 critical trials. Participants’ responses were scored as correct if they matched the pronunciation provided in the CTOPP manual, and incorrect otherwise. No partial credit was awarded.

##### Pseudoword repetition

The pseudoword repetition task, following Gupta (2003), asks participants to listen to a non-word and immediately repeat it back. Materials, taken from Gupta (2003), were created by combining syllables from English words into novel, phonotactically legal strings, such as *waydish* and *spentonymidderoxing*. After completing six practice trials, participants were given 96 items of either two, four, or seven syllables. Participants received credit for how many correctly-pronounced syllables they produced before making an error.

#### Perceptual Speed

Measures of perceptual speed were included in order to address concerns that individuals with more language experience are faster at the task overall due to a domain-general ability to process perceptual stimuli quickly, which could independently enhance reading skill and the ability to search a visual display for objects of interest. We included two measures of perceptual speed.

##### Letter comparison

In the letter comparison task, following Salthouse & Babcock (1991), participants were asked to compare two arrays of consonant letters as quickly as possible. Trials were presented in six blocks: two blocks comparing three-letter arrays, two blocks comparing six-letter arrays, and two blocks comparing nine-letter arrays. During each block, participants were given 20 seconds to complete as many comparison trials as possible. On all mismatching trials, only one letter differed between the arrays. Participants completed two practice trials with feedback, each with three-letter arrays, in which one trial contained a match and the other contained a mismatch.

##### Pattern comparison

The procedure of the pattern comparison task was the same as for letter comparison, except that arrays of line segments rather than letters are compared (Salthouse & Babcock, 1991). Blocks of three-, six-, and nine-segment arrays were presented in an order identical to that in the letter comparison task. After completing one match and one mismatch practice trial, participants were asked to perform the critical trials as quickly as possible.

#### Working memory

While performance on speeded tasks is a straightforward way to operationalize processing efficiency, the ability to hold multiple items in mind during demanding tasks is another test of efficiency. Working memory has played a prominent role in the investigation of individual differences in sentence processing, although much of this literature deals with complex syntactic structures (e.g. long distance dependencies in Gibson, 2000). Individuals who fixate the target more quickly may do so because they can effectively hold the sentential context in mind and make inferences about it before the sentence has concluded. Working memory was assessed using three complex span tasks, described in more detail below.

##### Reading span

The reading span task, adapted from Daneman and Carpenter (1980), required participants to read sentences out loud and make a judgment about whether the sentence was true. Sentences were taken from Stine and Hindman (1994). After the judgment was made, a single letter was displayed for the participant to remember, following Unsworth, Heitz, Schrock, and Engle (2005). While other versions of the task require participants to remember the final word of each sentence, a random letter was used so that participants’ memory performance would be less likely to be confounded with their skill at reading the sentences, or familiarity with the sentence-final words. To further correct for overall differences in reading ability, participants completed a calibration phase at the beginning of the task that excluded the letter-memory component. This determined how long they would be given to read the sentences during the test phase (Unsworth et al., 2005). Participants were then given two practice trials, each containing two sentence-letter pairs (i.e. a span length of two). The test trials then tested span lengths two to six in a random, rather than ascending order. The random presentation of all span lengths was done to gather information on the subject’s ability at each level (rather than stopping once they fail a span length, as is often done; see Conway, Kane, Bunting, Hambrick, Wilhelm, & Engle, 2005 for discussion of span task procedures). A second reason to randomize presentation is to deconfound span length with the increasing likelihood of proactive interference over time (Lustig, May & Hasher, 2001).

##### Listening span

The listening span task followed the same procedure as above, except that the sentences and letters were presented auditorily and the calibration phase was based on the latency to make the true/false judgment. No sentences during this phase were repeated from the reading span task, although they did also come from Stine and Hindman (1994).

##### Operation span

The operation span task procedure was similar to the reading and listening span. Rather than comprehend sentences, participants were asked to complete and verify math equations involving two operations. Each equation included either multiplication or division followed by either addition or subtraction. Once participants solved the problem, they pressed a spacebar to see a probe number, and participants indicated whether or not it was the correct solution to the preceding problem. As in the previous span tasks, participants completed a calibration phase that determined the maximum time they were permitted to spend on the processing portion of the task. After the judgment was complete, participants were presented with a letter to recall later, as in the previous tasks.

#### Inhibitory control

The final construct of interest captures part of the complex ability to allocate attention effectively during cognitive tasks. Inhibitory control is a domain-general ability falling under the general umbrella of executive attention. Inhibitory control is typically used to describe the ability to resist distraction from either internal or external stimuli, although Friedman and Miyake (2004) point out that definitions have been vague and inconsistently applied across literatures. Here, we conceptualize inhibitory control as the ability to override a conflicting response in favor of responding according to task goals.

It should also be noted that the complex span tasks described above can be conceptualized as measures of inhibitory control processes (Chun et al., 2011; Conway et al., 2005). This is true of other tasks, such as the n-back, which is treated as an inhibitory control measure and a working memory capacity measure (Kane & Engle 2002; Conway et al., 2005; Hussey & Novick, 2012). Inhibitory control as treated here is more specifically a measure of conflict resolution, or the ability to override salient cues or prepotent responses in favor of task-relevant information and responses. Conflict resolution in particular has played an important role in investigations of individual differences in sentence processing, specifically in ambiguity resolution and garden-path recovery (e.g. Gernsbacher, 1993, 1995; Novick et al., 2005, 2010). Even so, based on previous research on the relation between working memory and inhibitory control, we expect the tasks described here to be correlated with our measures of working memory (antisaccade: Kane et al., 2001; Unsworth, Schrock, & Engle, 2004; flanker: Heitz & Engle, 2007; Stroop: Kane & Engle, 2003).

##### Antisaccade

The antisaccade task requires the inhibition of a prepotent response to make a saccade to a suddenly presented stimulus in the visual field. Following Kane, Bleckley, Conway, and Engle (2001), participants began critical trials by fixating a plus sign in the center of the screen. An anti-predictive cue appeared at one side of the screen after a variable length of time to prevent participants from predicting when this cue would appear. A target letter (either B, R, or P) then appeared on the opposite side of the screen as the cue, preceded by a forward mask (the letter H) and followed by a backward mask (the number 8). Participants were asked to identify the target letter. Prior to the 72 critical trials, participants completed a response-mapping phase to learn which keys to press (1, 2, and 3) in response to the target letters, then 52 practice trials that gave a feedback tone only in response to incorrect responses.

##### Flanker

Participants completed a version of the “flankers” response competition paradigm (Eriksen & Eriksen, 1974; see Eriksen, 2007 for review) in which a visually-presented target item is flanked by either congruent items that facilitate correct responding, or incongruent items that inhibit correct responding. Participants in this task indicated the direction of an arrow that was flanked by four arrows of the same (< < < < <) or different (> > < > >) direction. The incongruent items are thought to activate the incorrect response, making it more difficult to select the correct response, as measured by response latency (Eriksen, 2007).

##### Stroop

Participants completed a self-paced version of the Stroop task (Stroop, 1935) in which they completed a conflict-free phase followed by a conflict phase. In both phases, the task is to name the color presented against a black background on the computer screen as quickly as possible. Participants were trained on the appropriate color names before the task. These were red, orange, yellow, green, blue, and purple. In the conflict-free phase, participants named aloud the color of a filled rectangle. In the conflict phase, the stimulus to be judged is a word, giving rise to a conflicting response of simply reading the word. The words were maximally conflicting, as they were task-relevant color terms that never matched the color that the stimulus was presented in (e.g. the word “blue” presented in green, where the correct response is to say green). Accuracy is typically high in the self-paced version of the task, so the difference in reaction time between the two phases was used as a measure of interference.

#### Eye-tracking

The design of the eye-tracking task is the same as described in Study 1. To increase statistical power, the number of predictive trials, neutral trials, and filler trials were each doubled, such that each participant completed 32 experimental trials and 32 filler trials. The additional materials for Study 2 are listed in Appendix A.

#### Procedure

As in Study 1, all participants completed all tasks in the same order. The individual differences battery comprised the 16 total tasks described above. The entire procedure took place over two sessions, scheduled 24 hours apart in order to minimize fatigue in each session. During the first session, participants completed an unrelated self-paced reading task (James et al., 2018), then the working memory tasks, the perceptual speed tasks, the inhibitory control tasks, and began the language experience tasks (ERVT and ART). The first session typically lasted 90–120 minutes. During the second session, participants completed the current eye-tracking task as well as another eye-tracking task that was part of a separate study. The procedure for the eye-tracking task is the same as the procedure described in Study 1. They then completed the three remaining language experience tasks and the phonological ability tasks. The session was concluded with a participant questionnaire and a debriefing. The second session typically lasted 40–60 minutes.

All individual differences tasks were programmed and displayed using the Matlab Psychophysics Toolbox (Brainard, 1997). Participants completed tasks at their own pace, without additional separation between the subcategories of tasks.

### Results

**Figure 5:**
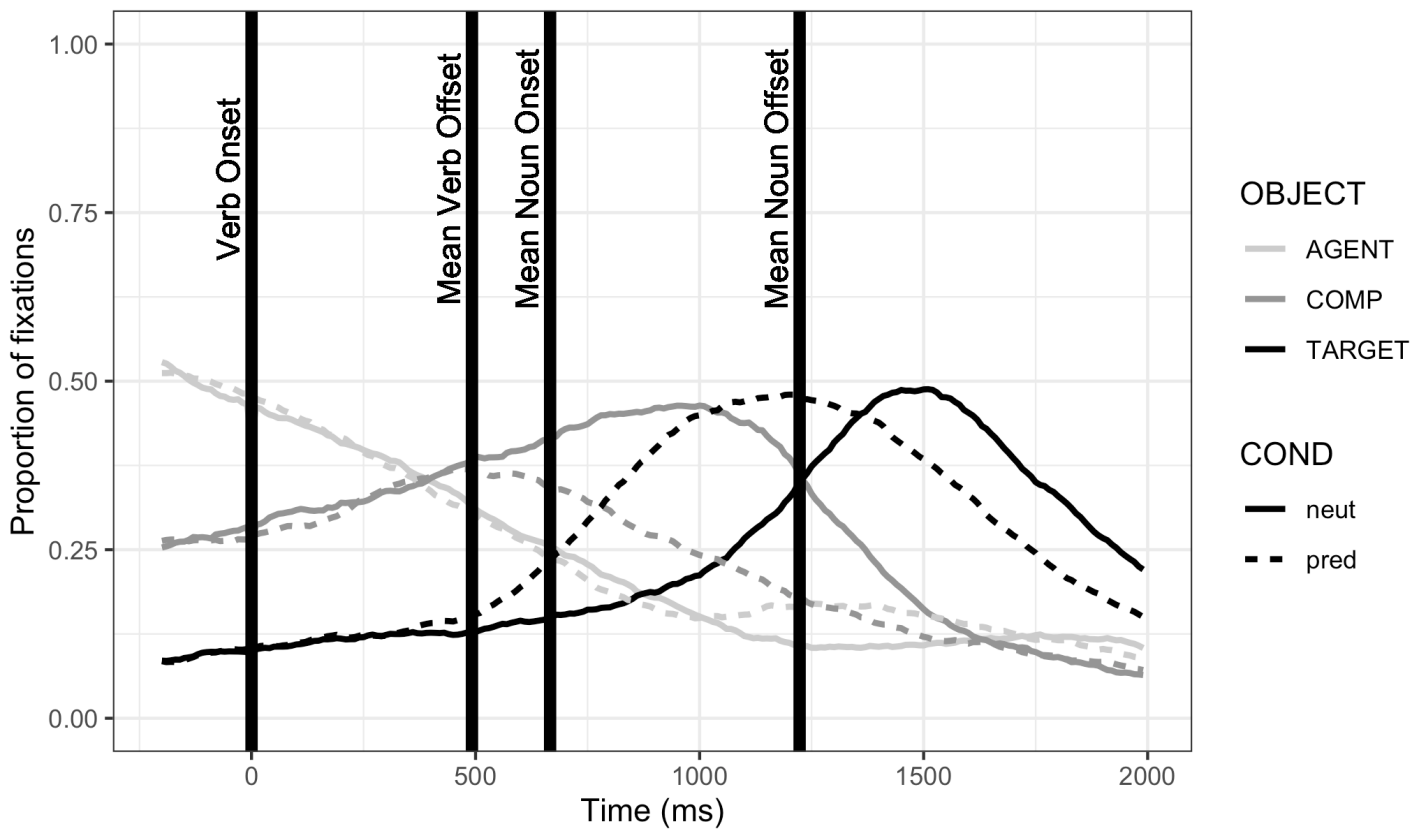
Proportion of fixation durations to regions of interest, by condition. *Note*: The y-axis presents the proportion of each 10-ms bin that was spent fixating the regions of interest (ROIs): the agent (e.g. the boy), the target (e.g. the cake), and any of the competitor objects (e.g. the sum of fixations to the car, ball, and train). Nonsense objects, which were included in half of critical trials, were included in the total of competitor fixations. The total proportion of fixations within a bin does not sum to one because of the time spent looking outside of the ROIs. The x-axis presents time starting from 200 ms before the verb onset, which is aligned at 0 ms; the means of verb offset and noun on- and offset times are shown for illustrative purposes.

**Figure 6:**
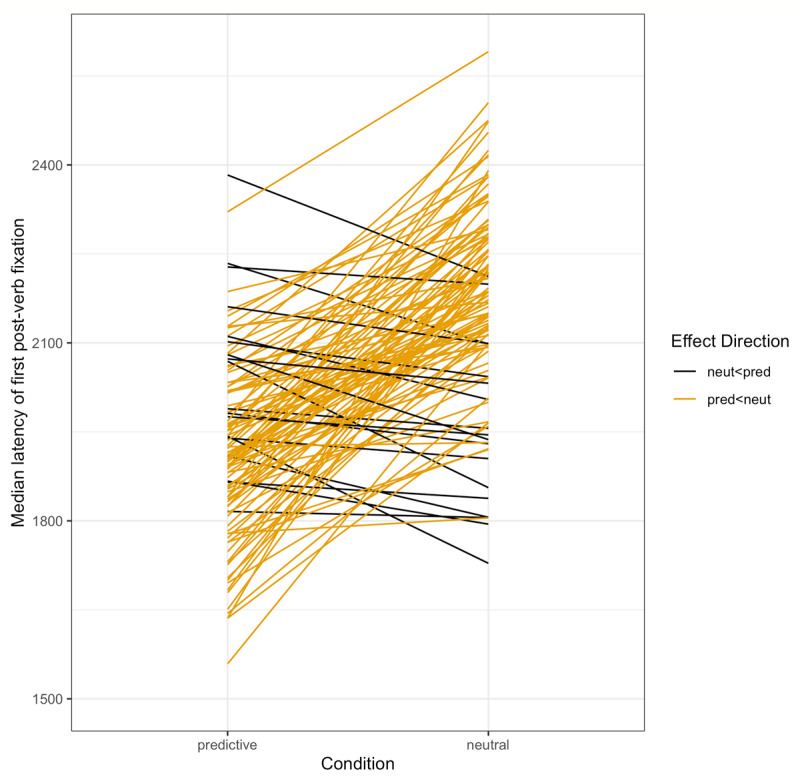
Study 2: Median latency to fixate the target by condition by subject. *Note*: Latencies here are much larger overall than in Study 1.

**Figure 7:**
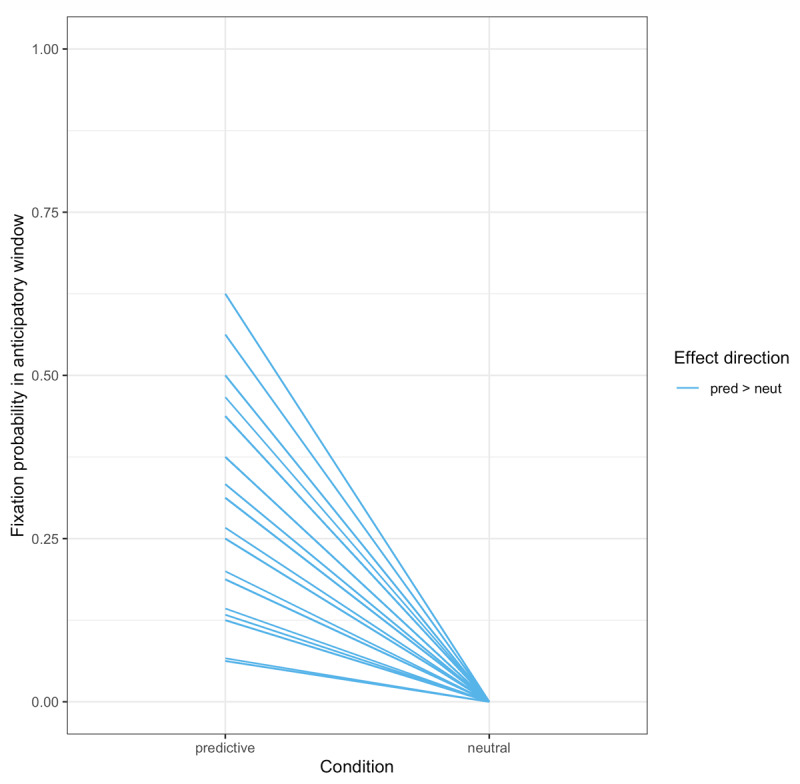
Study 2: Target fixation probability by condition by subject. *Note*: There were no target fixations in the neutral condition during the anticipatory window.

The eye-tracker failed to record 55 trials (0.73%). The eye-tracking data were analyzed as in Study 1. ROIs were defined by drawing a tight rectangle around each object; Figure 5 summarizes fixations to these ROIs over time. Again, our dependent measures were (a) latency of the first target fixation following the verb; and (b) whether the target was fixated during the anticipatory time window, bounded between the onset of the verb plus 200 ms, and the onset of the noun. Counts of trials with and without target fixations in the critical window, by condition, are given in Table 2. Notably, once target fixations that were initiated prior to the verb were removed, there were no neutral trials in which the target was fixated during the critical window; this led to analytical challenges (see Appendix B). Participant averages by condition for latency and fixation probability are given in Figures 6 and 7, respectively.

#### Analyses

All of the following analyses were performed using a Bayesian multilevel regression framework, as in Study 1. We obtain qualitatively similar results with traditional multilevel regression analyses, reported in Appendix B. All of the following models were built using the brms package (Bürkner, 2017) in RStudio (version 2022.07.2) and include the full random effects structure: random intercepts and slopes for both subjects and scenes. In all models, condition was dummy coded (neutral verb = 0, predictive verb = 1). Additional model specifications are described along with the model results, next.

##### Condition effect

First, we built a linear regression model predicting the log latency of the first post-verb fixation to the target object from condition. The latency model was constructed with weakly informative priors for the fixed effects and the brms default priors otherwise; two sampling chains ran for 8000 iterations. We replicated the effect of condition on latency, such that participants in the predictive condition were faster to fixate the target following the verb (estimate = –0.18, credible interval = [–0.30; –0.07], *p*(<0) = 0.999).

Next, we built a logistic regression model predicting whether the target was fixated in the anticipatory window. This model was constructed with weakly informative priors for the fixed effects and the default priors otherwise; two sampling chains ran for 10,000 iterations. Again, we replicated the condition effect, such that those in the predictive condition were more likely to fixate the target before hearing the noun.

##### Question 1: Reliability of individual differences in condition effect

Returning to our first research question, we ask whether there is evidence for individual differences in the condition effects. In Study 1, our data suggested that subjects had low internal consistency, and so we doubled the number of critical trials in an attempt to increase the precision of subjects’ estimated condition effects. Model comparisons suggested that random slopes for subjects were not justified in either the latency model (BF = 0.021) or the fixation probability model (BF = 0.177). On the other hand, a latency model with random intercepts was preferred (BF = 12.13). Unlike Study 1, we did not find support for random intercepts in the fixation probability model (BF = 0.084).

Repeating our split-half procedure from Study 1, the mean estimated internal consistency of the condition difference was very low for latency (mean = –0.07, SD = 0.15). The estimate for the probability of target fixations was 0.30 (SD = 0.09), but it is crucial to note that the probability of target fixations was always 0 in the neutral condition, so this estimate is equivalent to the consistency of fixation probabilities in the predictive condition.

Therefore, as in Study 1, we failed to provide evidence of stable individual differences in anticipatory eye movements, although there do seem to be baseline differences in the dependent measures.

The individual differences battery. Overall performance on the language experience tasks was similar performance in Study 1 (Table 7). Repeating the split-half procedure for the language experience measures resulted in mean correlations of 0.72 (SD = 0.05) for ART, 0.74 (SD = 0.04) for ERVT, and 0.74 (SD = 0.04) for NAART. The correlations among survey items within the CRH and RTE are given in Tables 8 and 9, respectively. Table 10 provides the correlations among language experience scores.

**Table 7:** Study 2 Individual Difference Battery Performance.


DOMAIN	TASK	POSSIBLE RANGE	OBSERVED RANGE	MEAN (SD)

Language experience	1 ART	[–65, 65]	[–9; 42]	10.44 (11.40)

	2 NAART	[0; 1]^a^	[0.30; 0.89]	0.56 (0.12)

	3 ERVT	[0; 48]	[1.25; 36.75]	17.36 (7.23)

	4 CRH	[7; 35]	[9; 33]	22.13 (5.09)

	5 RTE	[0; 63]	[5; 63]	20.37 (10.95)

Working memory	6 RSpan	[0;10]	[2.78; 10]	6.77 (1.72)

	7 LSpan	[0;10]	[5.63; 10]	8.91 (0.96)

	8 OSpan	[0;15]	[1.85; 14.83]	10.61 (3.45)

Inhibitory control^b^	9 AntiSac	n/a	[–1.97; 0.27]	–0.55 (0.31)

	10 Flanker	n/a	[–0.26; 0.34]	0.15 (0.07)

	11 Stroop	n/a	[–0.15; 0.51]	0.18 (0.14)

Phon. ability	12 Pseudo	[0; 1]^c^	[0.62; 0.95]	0.80 (0.07)

	13 BNW	[0; 1]^d^	[0.13; 0.96]	0.65 (0.16)

	14 PR	[0; 1]^d^	[0.14; 1]	0.69 (0.15)

Perceptual speed^e^	15 LComp	0+	[41; 120]	70.15 (14.25)

	16 PComp	0+	[46; 109]	81.12 (13.48)


*Notes*: ^a^ Proportion correct of 61 items. ^b^ Scores are log interference scores (RT difference); ranges are not meaningful here. ^c^ Proportion correct of 416 syllables across 96 items. ^d^ Proportion correct of 18 items. ^e^ Participants complete as many items as possible in six 20-second blocks; the final score is the total of correct items.

**Table 8:** Correlations Among Comparative Reading Habits Items in Study 2.


	TIME	COMPLEX	ENJOY	UNDERSTAND

Complex	0.568**	–		

Enjoy	0.619***	0.446***	–	

Speed	0.321**	0.226*	0.354***	–

Understand	0.457***	0.338***	0.249*	0.437***


*Note*: Participants answered five questions in which they compared themselves with their peers on how much time they spend reading (“Tme”), how complex their reading material is (“Complex”), how much they enjoy reading (“Enjoy”), how fast they read (“speed”), and how well they understand the material when reading at their normal pace (“Understand”). + *p* < 0.1. * *p* < 0.05. ** *p* < 0.01. *** *p* < 0.001.

**Table 9:** Correlations Among Reading Time Estimate Items in Study 2.


	1	2	3	4	5	6	7	8

1. Textbooks	–							

2. Academic texts^a^	0.51***	–						

3. Magazines	0.24*	0.25*	–					

4. Newspapers	0.25*	0.39**	0.62***	–				

5. Emails	0.24*	0.42***	0.50***	0.50***	–			

6. Websites^b^	0.01	0.21*	0.30**	0.40***	0.44***	–		

7. Fiction	0.11	0.15	0.37***	0.39***	0.36***	0.38***	–	

8. Non-fiction	0.37***	0.46***	0.39***	0.49***	0.40***	0.33***	0.57***	–

9. Other	0.23*	0.37***	0.47***	0.53***	0.46***	0.40***	0.54***	0.57***


*Note*: Participants estimated how much time they spent reading different types of material in a typical week. + *p* < 0.1. * *p* < 0.05. ** *p* < 0.01. *** *p* < 0.001. ^a^ Other than textbooks. ^b^ Other than email.

**Table 10:** Correlations Among Language Experience Task Scores in Study 2.


	ART	NAART	ERVT	CRH

NAART	0.39***	–		

ERVT	0.45***	0.68***	–	

CRH	0.25**	0.26**	0.35***	–

RTE	0.194*	0.182^+^	0.10	0.387***


*Note*: ART = Author Recognition Test, NAART = North American Adult Reading Test, ERVT = Extended Range Vocabulary Test, CRH = Comparative Reading Habits, RTE = Reading Time Estimate. + *p* < 0.1. * *p* < 0.05. ** *p* < 0.01. *** *p* < 0.001.

Performance on each of the tasks is given in Table 7. As in Study 1, we created composite scores by taking the mean of standardized task scores within each construct; correlations across constructs are given in Table 11.

**Table 11:** Study 2 Correlations Among Composite Scores.


TASK	1	2	3	4	5

1 Language experience	–				

2 Working memory	0.16^+^	–			

3 Inhibitory control	–0.05	–0.1	–		

4 Phonological ability	0.33***	0.30***	–0.04	–	

5 Perceptual speed	0.13	0.09	–0.28***	0.11*	–


*Note*: + *p* < 0.1. * *p* < 0.05. ** *p* < 0.01. *** *p* < 0.001.

##### Question 2: Language experience

Given the low within-subject internal consistency of the condition effects, it should be unsurprising to find no evidence of interactions between condition and any of the five composite scores in our models. On the other hand, our higher internal consistency in overall target fixations enables us to ask whether we replicate the main effect of language experience from Study 1. We added the language experience composite score and its interaction with condition as fixed effects, added weakly informative priors for those effects, and otherwise kept all specifications the same as in the condition-only models. Here, we see some evidence for a main effect of language experience on overall latency (estimate = –0.03, 95%–CI = [–0.07; 0.01], *p*(<0) = 0.907) but not probability of fixation (estimate = –3.21, 95%–CI = [–28.77; 10.53], *p*(>0) = 0.361). However, it is worth noting that there is less evidence for a language main effect on latency here than in Study 1, with a lower proportion of posterior estimates at 0 or higher (0.907 in Study 2 vs. 0.978 in Study 1).

##### Question 3: Controlling for other cognitive predictors

Finally, we asked whether the main effect of language experience in the latency model remained after we added our other measures of cognitive skills. Starting from the language experience and condition model described above, we added fixed effects for verbal working memory, inhibitory control, phonological ability, and perceptual speed composite scores, as well as each of their interactions with condition. We also specified weakly informative priors for each of the fixed effects. Otherwise, model specifications were the same as in the previous models. The main effect of language experience in predicting latency was maintained (estimate = –0.04, 95%–CI = [–0.08; 0.01], *p*(<0) = 0.946).

### Discussion

In summary, Study 2 replicated the pattern of results from Study 1: across subjects, there was strong evidence for an effect of verb condition on eye movements; there was low within-subject consistency in the size of those condition effects; internal consistency was higher in subjects’ overall latency to fixate the target; and our language experience composite scores predicted differences in overall latency. Further, Study 2 expanded on those findings by demonstrating substantial evidence that the language experience relation survives the introduction of other constructs that could reasonably have explained it. In the General Discussion that follows, we unpack these findings in light of theoretical, methodological, and statistical considerations.

## General Discussion

We began by introducing three central research questions about the nature of language-mediated anticipatory eye movements. Taking both studies together, we have a consistent pattern of results that address each question: (1) evidence for individual differences in language-mediated eye movements appears in overall fixation latencies but not *condition effects* on latencies or on the probability of fixating the target in the anticipatory time window; (2) evidence that our measure of language experience, a composite of five tasks, was related to overall fixation latencies; and (3) evidence that this relationship was upheld when working memory, inhibitory control, phonological ability, and perceptual speed were introduced as predictors. We next take each of these findings in turn.

### Question 1: Individual differences in language-mediated eye movements

We set out to use individual differences as a tool to investigate the relation between language experience and online auditory sentence processing. However, such an investigation depends on the existence of robust individual differences in the first place. There has been increasing evidence in the cognitive psychology literature generally, and with respect to online language processing specifically, that stable individual differences cannot be assumed. In fact, Hedge and colleagues (2018) demonstrate, using the domain of executive control tasks, that more robust effects (averaged over subjects) tend to be those with *less* between-subject variability. A possibility is that this anticipation effect, which has been replicated in adult native speakers many times (see Kamide, 2008 and Huettig et al., 2011 for reviews), is not an ideal candidate for investigating individual differences.

However, there are a number of methodological considerations that likely played a role in our difficulty in estimating subject-level eye movement performance in this task. First, there was substantial item variability, which compromised within-subject consistency. Following the original Altmann & Kamide (1999) visual displays, we varied the sizes and placements of objects to create a composed scene rather than create simpler displays with less variability in object characteristics, as in four-quadrant designs. Of particular importance to our fixation probability measure, our anticipatory window was very short, comprising only the critical verb and the determiner preceding the target noun. The anticipatory window could have been expanded by including neutral intervening information between verb and target noun (e.g. “The boy will eat/move the *very wonderful* cake). Other work has expanded the anticipatory window by allowing predictive information to accumulate from multiple words within the sentence (e.g. Borosky et al. 2012: “The *dog* will *bury* the bone”).

### Questions 2 and 3: Language experience link, beyond general cognitive skills

The current work suggests that language experience, as measured by our composite score, is related to language-mediated eye movements even when other cognitive skills are taken into account. This points to the ability of language experience to capture unique variability in spoken comprehension, even within a literate population that represents a restricted range of language ability.

Results from Mishra and colleagues (2012) and Borovsky and colleagues (2012) are consistent with our evidence for a more general benefit of experience. Highly literate adults showed facilitation relative to low-literate adults in trials that licensed anticipation, in line with the experience effect shown here. However, their studies did not include control trials without predictive information, and it is possible that the literate adults would have been facilitated on these trials as well. For instance, Mishra and colleagues suggest that individuals of higher literacy have “fine-tuned” their anticipatory mechanisms through practice with reading and writing. In light of our current evidence, it could be the case that this fine-tuning promotes efficient processing on the non-predictable trials as well. A more detailed account of how this happens is a topic for further study.

Individuals with more experience may be more likely to try to make sense of the visual scene, exploring the various objects to anticipate what will be referred to. They may be facilitated in processing the sentence as it unfolds due to bottom-up word recognition processes, and, having processed the unfolding sentence more easily, participants might have resources free to search for the upcoming target in the scene as the sentence continues. The current study found support for this in looks away from the agent, which was always the subject of the accompanying sentence (e.g. “The boy will…”). Individuals with more language experience look less at the agent while processing the verb across conditions (e.g. “eat/move”).

One potential concern is that individuals with more experience are simply more motivated to complete the task, and make more of an effort to find ways to anticipate the target. Under this hypothesis, the effect would go away if participants were no longer performing an explicit judgment task (although they may still implicitly consider other task goals; see Salverda, Brown, & Tanenhaus, 2011 for discussion). We find this explanation unlikely since highly motivated individuals would likely try harder (and be more likely to succeed) at the other measures, and we only found language experience task performance to correlate with phonological processing tasks, the other group of explicitly language-oriented tasks.

An important aspect of this work is the demonstration of a link between listening skills and literacy. However, the nature of this relationship is still a puzzle. One potential explanation for this link is that experience with reading benefits auditory comprehension by providing the processing system with information about the probabilities of the language, leading to efficient comprehension. This assumes that the language processing system applies knowledge gained in the written modality and applies it to the spoken domain. Of course, this explanation does not exclude the possibility that auditory experience and listening comprehension influence reading experience. Given that phonological ability both predicted eye movements and correlated with the language experience measures, it is possible that there is a link between reading and listening experience: phonological ability facilitates word recognition during listening, but also promotes efficient reading (Stanovich & West, 1989; MacDonald & Christiansen, 2002; Acheson & MacDonald, 2009; 2011). Efficient readers may then gain more reading experience (see Matthew effects discussed earlier; Stanovich, 1986), which provides a benefit in auditory processing over and above that provided by increased phonological skills.

### Conclusion

While questions remain open regarding the mechanism linking more experience to performance in this auditory task, the current work makes two important contributions to the study of individual differences in sentence processing. First, we found that experience with language, largely related to reading experience, predicts online performance in the auditory domain, which speaks to the potential general benefit of exposure to various linguistic contexts. Second, by measuring a variety of other constructs previously involved in individual differences research, we were able to demonstrate a benefit of exposure that goes beyond more general cognitive mechanisms. These results reinforce the importance of literacy education and ongoing growth in exposure to print, suggesting that reading skill influences listening skills into adulthood.

## Data Accessibility Statements

The results are kept in strict confidence, and are available to no one apart from those individuals immediately involved in the research project.

## Appendix A

### Study 1

Critical trials use the same sentences and distractor objects as in Altmann and Kamide (1999) unless otherwise noted (*), with the former version in brackets. Nonsensical items are indicated with italics.

**1.** The boy will eat/move the cake. (toy car, ball, toy train).
***2.** The woman will drink/try the wine. (cheese, lipstick, perfume [chair], *plant*).
**3.** The policeman will arrest/search the man. (car, dustbin, houses, *cat*).
**4.** The woman will bathe/touch the baby. (plant, rocking-horse, stool).
**5.** The boy will bounce/throw the ball. (paper plane, shuttle-cock, acorns, *bicycle*).
**6.** The hiker will climb/photograph the mountain. (mountain lion, moon, cactus).
***7.** The housewife will fry/grab [wash] the shrimp. (knife, jug, weighing scales).
**8.** The doctor will inject/check the child. (TV monitor, microscope, books, *toy bear*).
**9.** The woman will play/dust the piano. (table, television, telephone).
**10.** The woman will read/open the book. (door, bag, jar, *cup*).
***11.** The man will repair/wipe the washing machine. (mirror, goggles [waste bin], wrench [dog]).
**12.** The baby will ring/kick the bell. (drum, bricks, duck).
**13.** The man will sail/watch the boat. (birds, car, sun).
**14.** The man will smoke/collect the cigarettes. (binder, glasses, briefcase, *clock*).
**15.** The boy will walk/feed the dog. (bird, pig, hen, *ball*).
**16.** The businessman will wear/forget the hat. (wallet, folder, magnifying glass, *chair*).

### Study 2

Sentences and distractor objects for the filler trials were invented for the current study. Following Altmann and Kamide (1999), they were designed such that either none, one, two, or three of the items in the scene made sense given the preceding context. Nonsensical items are indicated will italics. As in the critical scenes, there were either four or five objects in the scene and an agent.

**1.** The baby will lift the rattle (*rocking chair, grandfather clock, bureau, trunk*)
**2.** The woman will rip the page (*ice cream, egg, phone, bowling ball*)
**3.** The woman will sew the dress (*stool, clock, laptop, tomato, hat box*)
**4.** The man will shut the window (*puppy, vacuum, grapes, pumpkin, oven mitt*)
**5.** The man will shake the maraca (salt, *toaster, cheese grater, coffee maker*)
**6.** The maid will fold the slacks (shirt, *cup, stapler, vase*)
**7.** The man will stir the coffee (soup, *banana, money, tissue box, lamp*)
**8.** The child will taste the candy (cookie, *yo-yo, teddy, bench, parrot*)
**9.** The girl will ride the unicycle (horse, scooter, *shrub, football*)
**10.** The teenager will zip the hoodie (boot, jacket, *sandwich, newspaper*)
**11.** The silversmith will polish the spoon (teapot, watch, *child, couch, pillow*)
**12.** The man will shatter the vase (jar, bottle, *hotdog, purse, onion*)
**13.** The farmer will hear the siren (rooster, telephone, radio, *bread*)
**14.** The girl will pet the puppy (kitten, bunny, hamster, *pie*)
**15.** The musician will tune the saxophone (violin, guitar, banjo, *cabbage, bag*)
**16.** The man will sip the martini (tea, milkshake, beer, *candle, basket*)

## Appendix B

We present here the results of our original analyses, which were performed in RStudio (Workbench 2021.09.2 + 382.pro1) with lmerTest (version 3.1–3; Kuznetsova et al., 2017). For the sake of simplicity, we present only the models of condition with language experience (Study 1, Question 2) and will all individual difference constructs (Study 2, Question 3), as they contain all of the critical fixed effects of interest.

### Model selection

We started with a random effects structure that included random intercepts and slopes for both subjects and scenes, and then removed random effects until the resulting model (1) did not have a singular fit and (2) was preferred to a more complex model according to a likelihood ratio test using the lrtest function in the lmtest package (version 0.9–40, Zeileis & Hothorn, 2002; see Matuschek, Kliegl, Vasishth, Baayen, & Bates, 2017 for discussion of model selection).

### Study 1

In predicting log-transformed latencies, our model selection procedure resulted in a model with random intercepts for subjects and scenes, and random slopes only for scenes. The model of fixation probability only included random intercepts. In predicting both latency and fixation probability, the verb condition had a significant effect on the outcome (Latency: \hat \beta = –0.19, *SE* = 0.040, *t* = –4.721, *p* < 0.001; Probability: \hat \beta = 0.54, *SE* = 0.114, *z* = 4.744, *p* < 0.001). The language-by-condition interaction was not statistically significant for either outcome (Latency: \hat \beta = 0.020, *SE* = 0.036, *t* = 0.554, *p* = 0.580; Probability: \hat \beta = 0.007, *SE* = 0.166, *z* = 0.045, *p* = 0.964). However, for latency, language experience ( \hat \beta = –0.06, *SE* = 0.030, *t* = –2.002, *p* = 0.046), such that higher composite scores predicted faster target latencies across verb conditions. There was no such effect for fixation probability ( \hat \beta = 0.194, *SE* = 0.145, *z* = 1.342, *p* = 0.180).

### Study 2

In predicting latency, our model selection procedure resulted in a model with random intercepts only. We predicted log-transformed latencies from condition, each of the five composite scores, and the interactions between condition and each of these. Again, there was a robust effect of condition ( \hat \beta = –0.19, *SE* = 0.040, *t* = –3.346, *p* < 0.001). The main effect of language experience was marginal ( \hat \beta = –0.04, *SE* = 0.022, *t* = –1.651, *p* = 0.096). By comparison, when the same model was run with only language experience (excluding the four other constructs), the language main effect was not statistically significant (*p* = 0.16).

We were unable to fit models of fixation probabilities because there were no positive cases (trials with any fixations to the target) in the neutral condition.

## References

[1] Acheson, D. J., & MacDonald, M. C. (2009). Verbal working memory and language production: Common approaches to the serial ordering of verbal information. Psychological Bulletin, 135, 50–68. DOI: 10.1037/a0014411 19210053 PMC3000524

[2] Acheson, D. J., & MacDonald, M. C. (2011). The rhymes that the reader perused confused the meaning: Phonological effects during on-line sentence comprehension. Journal of Memory and Language, 65, 193–207. DOI: 10.1016/j.jml.2011.04.006 21743771 PMC3129981

[3] Acheson, D. J., Wells, J. B., & MacDonald, M. C. (2008). New and updated tests of print exposure and reading abilities in college students. Behavior Research Methods, 40(1), 278–289. DOI: 10.3758/BRM.40.1.278 18411551 PMC3022331

[4] Allopenna, P. D., Magnuson, J. S., & Tanenhaus, M. K. (1998). Tracking the time course of spoken word recognition using eye movements: Evidence for continuous mapping models. Journal of Memory and Language, 38(4), 419–439. DOI: 10.1006/jmla.1997.2558

[5] Altmann, G., & Kamide, Y. (1999). Incremental interpretation at verbs: Restricting the domain of subsequent reference. Cognition, 73(3), 247–264. DOI: 10.1016/S0010-0277(99)00059-1 10585516

[6] Barr, D.J., Levy, R., Scheepers, C., & Tilly, H. J. (2013). Random effects structure for confirmatory hypothesis testing: Keep it maximal. Journal of Memory and Language, 68, 255–278. DOI: 10.1016/j.jml.2012.11.001 PMC3881361 24403724

[7] Bates, D., Mächler, M., Bolker, B., & Walker, S. (2015). Fitting linear mixed-effects models using lme4. Journal of Statistical Software, 67(1), 1–48. DOI: 10.18637/jss.v067.i01

[8] Blair, J. R., & Spreen, O. (1989). Predicting premorbid IQ: a revision of the National Adult Reading Test. The Clinical Neuropsychologist, 3(2), 129–136. DOI: 10.1080/13854048908403285

[9] Borovsky, A., Elman, J. L., & Fernald, A. (2012). Knowing a lot for one’s age: Vocabulary skill and not age is associated with anticipatory incremental sentence interpretation in children and adults. Journal of Experimental Child Psychology, 112(4), 417–436. DOI: 10.1016/j.jecp.2012.01.005 22632758 PMC3374638

[10] Brainard, D. H. (1997). The Psychophysics Toolbox, Spatial Vision, 10, 433–436. DOI: 10.1163/156856897X00357 9176952

[11] Bürkner, P. C. (2017). brms: An R package for Bayesian multilevel models using Stan. Journal of Statistical Software, 80, 1–28. DOI: 10.18637/jss.v080.i01

[12] Chun, M. M. (2011). Visual working memory as visual attention sustained internally over time. Neuropsychologia, 49(6), 1407–1409. DOI: 10.1016/j.neuropsychologia.2011.01.029 21295047

[13] Conway, A. R., Kane, M. J., Bunting, M. F., Hambrick, D. Z., Wilhelm, O., & Engle, R. W. (2005). Working memory span tasks: A methodological review and user’s guide. Psychonomic Bulletin & Review, 12, 769–786. DOI: 10.3758/BF03196772 16523997

[14] Cooper, R. M. (1974). The control of eye fixation by the meaning of spoken language: A new methodology for the real-time investigation of speech perception, memory, and language processing. Cognitive Psychology 6(1), 84–107. DOI: 10.1016/0010-0285(74)90005-X

[15] Corsi, P. M. (1972). Human memory and the medial temporal region of the brain. [Doctoral dissertation, McGill University]. eScholarship @ McGill. https://escholarship.mcgill.ca/concern/theses/05741s554

[16] Cronbach, L. J. (1957). The two disciplines of scientific psychology. American Psychologist, 12, 671–684. DOI: 10.1037/h0043943

[17] Daneman, M., & Carpenter, P. A. (1980). Individual differences in working memory and reading. Journal of Verbal Learning and Verbal Behavior, 19, 450–466. DOI: 10.1016/S0022-5371(80)90312-6

[18] Ekstrom, R. B., French, J. W., Harman, H. H., & Dermen, D. (1976). Manual for kit of factor-referenced cognitive tests. Princeton, NJ: Educational Testing Service. Retrieved from http://www.ets.org/Media/Research/pdf/Manual_for_Kit_of_Factor-Referenced_Cognitive_Tests.pdf

[19] Eriksen, B. A., & Eriksen, C. W. (1974). Effects of noise letters upon the identification of a target letter in a nonsearch task. Perception & Psychophysics, 16(1), 143–149. DOI: 10.3758/BF03203267

[20] Eriksen, C. W. (2007). The flankers task and response competition: A useful tool for investigating a variety of cognitive problems. Visual Cognition, 2(2–3), 101–118. DOI: 10.1080/13506289508401726

[21] Farris-Trimble, A., & McMurray, B. (2013). Test-retest reliability of eye tracking in the visual world paradigm for the study of real-time spoken word recognition. Journal of Speech, Language, and Hearing Research, 56(4), 1328–45. DOI: 10.1044/1092-4388(2012/12-0145) PMC3875834 23926331

[22] Fernald, A., Perfors, A., & Marchman, V. A. (2006). Picking up speed in understanding: Speech processing efficiency and vocabulary growth across the 2nd year. Developmental Psychology, 42(1), 98–116. DOI: 10.1037/0012-1649.42.1.98 16420121 PMC3214591

[23] Fernald, A., Pinto, J. P., Swingley, D., Weinberg, A., & McRoberts, G. W. (1998). Rapid gains in speed of verbal processing by infants in the 2nd year. Psychological Science, 9(3), 228–231. DOI: 10.1111/1467-9280.00044

[24] Friedman, N. P., & Miyake, A. (2004). The relations among inhibition and interference control functions: A latent-variable analysis. Journal of Experimental Psychology: General, 133(1), 101–135. DOI: 10.1037/0096-3445.133.1.101 14979754

[25] Gernsbacher, M. A. (1993). Less skilled readers have less efficient suppression mechanisms. Psychological Science, 4, 294–298. DOI: 10.1111/j.1467-9280.1993.tb00567.x 25309046 PMC4191741

[26] Gernsbacher, M. A. (1995). The Structure-Building Framework: What it is, what it might also be, and why. In B. K. Britton, & A. C. Graesser, (Eds.), Models of text understanding (pp. 289–311). Hillsdale, NJ: Erlbaum.

[27] Gibson, E. (2000). The dependency locality theory: A distance-based theory of linguistic complexity. In A. Marantz, Y. Miyashita, & W. O’Neil (Eds.), Image, Language, Brain: Papers from the First Mind Articulation Project Symposium (pp. 94–126). Cambridge, MA: The MIT Press.

[28] Gupta, P. (2003). Examining the relationship between word learning, nonword repetition, and immediate serial recall in adults. The Quarterly Journal of Experimental Psychology, 56A, 1213–1236. DOI: 10.1080/02724980343000071 12959911

[29] Hedge, C., Powell, G., & Sumner, P. (2018). The reliability paradox: Why robust cognitive tasks do not produce reliable individual differences. Behavior Research Methods, 50, 1166–1186. DOI: 10.3758/s13428-017-0935-1 28726177 PMC5990556

[30] Heitz, R. P., & Engle, R. W. (2007). Focusing the spotlight: Individual differences in visual attention control. Journal of Experimental Psychology: General, 136(2), 217. DOI: 10.1037/0096-3445.136.2.217 17500647

[31] Hintz, F., Meyer, A. S., & Huettig, F. (2017). Predictors of verb-mediated anticipatory eye movements in the visual world. Journal of Experimental Psychology: Learning, Memory, and Cognition, 43(9), 1352–1374. DOI: 10.1037/xlm0000388 28287762

[32] Huettig, F., Singh, N., & Mishra, R. K. (2011). Language-mediated visual orienting behavior in low and high literates. Frontiers in Psychology, 2, 285. DOI: 10.3389/fpsyg.2011.00285 22059083 PMC3203553

[33] Huettig, F., Rommers, J., & Meyer, A. S. (2011). Using the visual world paradigm to study language processing: A review and critical evaluation. Acta Psychologica, 137(2), 151–171. DOI: 10.1016/j.actpsy.2010.11.003 21288498

[34] Huettig, F., & Janse, E. (2016). Individual differences in working memory and processing speed predict anticipatory spoken language processing in the visual world. Language, Cognition and Neuroscience, 31(1), 80–93. DOI: 10.1080/23273798.2015.1047459

[35] Hussey, E. K., & Novick, J. M. (2012). The benefits of executive control training and the implications for language processing. Frontiers in Psychology, 3, 158. DOI: 10.3389/fpsyg.2012.00158 22661962 PMC3356880

[36] James, A. N., Fraundorf, S. H., Lee, E. K., & Watson, D. G. (2018). Individual differences in syntactic processing: Is there evidence for reader-text interactions? Journal of Memory and Language, 102, 155–181. DOI: 10.1016/j.jml.2018.05.006 30713367 PMC6350810

[37] Kamide, Y. (2008). Anticipatory processes in sentence processing. Language and Linguistics Compass, 2(4), 647–670. DOI: 10.1111/j.1749-818X.2008.00072.x

[38] Kamide, Y., Altmann, G. T., & Haywood, S. L. (2003). The time-course of prediction in incremental sentence processing: Evidence from anticipatory eye movements. Journal of Memory and Language, 49(1), 133–156. DOI: 10.1016/S0749-596X(03)00023-8

[39] Kane, M. J., Bleckley, M. K., Conway, A. R. A., & Engle, R. W. (2001). A controlled-attention view of working-memory capacity. Journal of Experimental Psychology: General, 130, 169–183. DOI: 10.1037//0096-3445.130.2.169 11409097

[40] Kane, M. J., & Engle, R. W. (2002). The role of prefrontal cortex in working-memory capacity, executive attention, and general fluid intelligence: An individual-differences perspective. Psychonomic bulletin & review, 9(4), 637–671. DOI: 10.3758/BF03196323 12613671

[41] Kane, M. J., & Engle, R. W. (2003). Working-memory capacity and the control of attention: The contributions of goal neglect, response competition, and task set to Stroop interference. Journal of Experimental Psychology: General, 132(1), 47. DOI: 10.1037/0096-3445.132.1.47 12656297

[42] Kruschke, J. K., & Liddell, T. M. (2018). The Bayesian New Statistics: Hypothesis testing, estimation, meta-analysis, and power analysis from a Bayesian perspective. Psychonomic Bulletin & Review, 25, 178–206. DOI: 10.3758/s13423-016-1221-4 28176294

[43] Kuznetsova, A., Brockhoff, P. B., Christensen, R. H. B. (2017). lmerTest package: Tests in linear mixed effects models. Journal of Statistical Software, 82(13), 1–26. DOI: 10.18637/jss.v082.i13

[44] Lustig, C., May, C. P., & Hasher, L. (2001). Working memory span and the role of proactive interference. Journal of Experimental Psychology: General, 130(2), 199–207. DOI: 10.1037/0096-3445.130.2.199 11409099

[45] MacDonald, M. C., & Christiansen, M. H. (2002). Reassessing working memory: Comments on Just and Carpenter (1992) and Waters and Caplan (1996). Psychological Review, 109, 35–54. DOI: 10.1037/0033-295X.109.1.35 11863041

[46] Mani, N., & Huettig, F. (2012). Prediction during language processing is a piece of cake—But only for skilled producers. Journal of Experimental Psychology: Human Perception and Performance, 38(4), 843. DOI: 10.1037/a0029284 22774799

[47] Mani, N., & Huettig, F. (2014). Word reading skill predicts anticipation of upcoming spoken language input: A study of children developing proficiency in reading. Journal of Experimental Child Psychology, 126, 264–279. DOI: 10.1016/j.jecp.2014.05.004 24955519

[48] Matuschek, H., Kliegl, R., Vasishth, S., Baayen, H., & Bates, D. (2017). Balancing Type I error and power in linear mixed models. Journal of Memory and Language, 94, 305–315. DOI: 10.1016/j.jml.2017.01.001

[49] Merriam-Webster.com. Retrieved 2012, from http://www.merriam-webster.com/dictionary

[50] Mishra, R., Singh, N., Pandey, A., & Huettig, F. (2012). Spoken language-mediated anticipatory eye movements are modulated by reading ability: Evidence from Indian low and high literates. Journal of Eye Movement Research, 5(1), 3, 1–10. DOI: 10.16910/jemr.5.1.3

[51] Nelson, H. E. (1982). National Adult Reading Test (NART): Test Manual. Windsor: NFER-NELSON.

[52] Novick, J. M., Trueswell, J. C., & Thompson-Schill, S. L. (2005). Cognitive control and parsing: Reexamining the role of Broca’s area in sentence comprehension. Cognitive, Affective, & Behavioral Neuroscience, 5(3), 263–281. DOI: 10.3758/CABN.5.3.263 16396089

[53] Novick, J. M., Trueswell, J. C., & Thompson-Schill, S. L. (2010). Broca’s area and language processing: Evidence for the cognitive control connection. Language and Linguistics Compass, 4, 906–924. DOI: 10.1111/j.1749-818X.2010.00244.x

[54] Oberauer, K. (2022). The importance of random slopes in mixed models for Bayesian hypothesis testing. Psychological Science, 33(4), 648–665. DOI: 10.1177/09567976211046884 35357978

[55] Posner, M. I., Nissen, M. J., & Ogden, W. C. (1978). Modes of perceiving and processing information. Attended and unattended processing modes: the role of set for spatial location. In H. L. Pick & E. J. Saltzman (Eds.), Modes of Perceiving and Processing Information (pp. 137–157). Hillsdale, NJ: Erlbaum.

[56] Pronk, T., Molenaar, D., Wiers, R. W., & Murre, J. (2022). Methods to split cognitive task data for estimating split-half reliability: A comprehensive review and systematic assessment. Psychonomic Bulletin & Review, 29(1), 44–54. DOI: 10.3758/s13423-021-01948-3 34100223 PMC8858277

[57] Raven, J. C., & Court, J. H. (1998). Raven’s Progressive Matrices and Vocabulary Scales (pp. 223–237). Oxford: Oxford Psychologists Press.

[58] Rommers, J., Meyer, A. S., & Huettig, F. (2015). Verbal and nonverbal predictors of language-mediated anticipatory eye movements. Attention, Perception, & Psychophysics, 77, 720–730. DOI: 10.3758/s13414-015-0873-x 25795276

[59] Salthouse, T. A. (1996). The processing-speed theory of adult age differences in cognition. Psychological Review, 103(3), 403–428. DOI: 10.1037/0033-295X.103.3.403 8759042

[60] Salthouse, T. A., & Babcock, R. L. (1991). Decomposing adult age differences in working memory. Developmental Psychology, 27(5), 763. DOI: 10.1037/0012-1649.27.5.763

[61] Salverda, A. P., Brown, M., & Tanenhaus, M. K. (2011). A goal-based perspective on eye movements in visual world studies. Acta Psychologica, 137(2), 172–180. DOI: 10.1016/j.actpsy.2010.09.010 21067708 PMC3109199

[62] Sedivy, J. C., Tanenhaus, M. K., Chambers, C. G., & Carlson, G. N. (1999). Achieving incremental semantic interpretation through contextual representation. Cognition, 71(2), 109–147. DOI: 10.1016/S0010-0277(99)00025-6 10444906

[63] Smith, N. J., & Levy, R. (2013). The effect of word predictability on reading time is logarithmic. Cognition, 128(3), 302–319. DOI: 10.1016/j.cognition.2013.02.013 23747651 PMC3709001

[64] Smith, A. C., Monaghan, P., & Huettig, F. (2014). Literacy effects on language and vision: Emergent effects from an amodal shared resource (ASR) computational model. Cognitive Psychology, 75, 28–54. DOI: 10.1016/j.cogpsych.2014.07.002 25171049

[65] Stanovich, K. E., & West, R. F. (1989). Exposure to print and orthographic processing. Reading Research Quarterly, 24, 402–433. DOI: 10.2307/747605

[66] Stine, E. A. L., & Hindman, J. (1994). Age differences in reading time allocation for propositionally dense sentences. Aging and Cognition, 1, 2–16. DOI: 10.1080/09289919408251446

[67] Stroop, J. R. (1935). Studies of interference in serial verbal reactions. Journal of Experimental Psychology, 18, 643–662. DOI: 10.1037/h0054651

[68] Swets, B., Desmet, T., Hambrick, D. Z., & Ferreira, F. (2007). The role of working memory in syntactic ambiguity resolution: A psychometric approach. Journal of Experimental Psychology: General, 136(1), 64. DOI: 10.1037/0096-3445.136.1.64 17324085

[69] Tanenhaus, M. K., Spivey-Knowlton, M. J., Eberhard, K. M., & Sedivy, J. C. (1995). Integration of visual and linguistic information in spoken language comprehension. Science, 268(5217), 1632–1634. DOI: 10.1126/science.7777863 7777863

[70] Unsworth, N., Heitz, R. P., Schrock, J. C., & Engle, R. W. (2005). An automated version of the operation span task. Behavior Research Methods, 37, 498–505. DOI: 10.3758/BF03192720 16405146

[71] Unsworth, N., Schrock, J. C., & Engle, R. W. (2004). Working memory capacity and the antisaccade task: Individual differences in voluntary saccade control. Journal of Experimental Psychology: Learning, Memory, and Cognition, 30(6), 1302. DOI: 10.1037/0278-7393.30.6.1302 15521806

[72] Uttl, B. (2002). North American Adult Reading Test: Age norms, reliability, and validity. Journal of Clinical and Experimental Neuropsychology, 24(8), 1123–1137. DOI: 10.1076/jcen.24.8.1123.8375 12650237

[73] Viviani, P. (1990). Eye movements in visual search: Cognitive, perceptual, and motor control aspects. E. Kowler (Ed.), Eye movements and their role in visual and cognitive processes (Series: Reviews of Oculomotor Research, Volume 4). Elsevier: Amsterdam. 7492533

[74] Wagner, R. K., Torgesen, J. K., & Rashotte, C. A. (1999). Comprehensive test of phonological processing: CTOPP. Pro-Ed.

[75] Wechsler Adult Intelligence Test. (2004). Amsterdam. 3rd ed., Dutch version. The Netherlands: Harcourt Test.

[76] Westfall, J., & Yarkoni, T. (2016). Statistically controlling for confounding constructs is harder than you think. PloS one, 11(3), e0152719. DOI: 10.1371/journal.pone.0152719 27031707 PMC4816570

[77] Zeileis, A., & Hothorn, T. (2002). Diagnostic checking in regression relationships. R News, 2(3), 7–10. https://CRAN.R-project.org/doc/Rnews/

