# Correction: Language Experience Predicts Eye Movements During Online Auditory Comprehension

**DOI:** 10.5334/joc.354

**Published:** 2024-02-23

**Authors:** Ariel N. James, Colleen J. Minnihan, Duane G. Watson

**Affiliations:** 1 Department of Psychology, Macalester College, United States; 2 Department of Psychology and Human Development, Peabody College, Vanderbilt University, United States

**Keywords:** Eye movements, Sentence processing, Reading, Working memory, Cognitive Control, Auditory word processing

## Abstract

This article details a correction to: James, A.N., Minnihan, C.J. and Watson, D.G., 2023. Language Experience Predicts Eye Movements During Online Auditory Comprehension. Journal of Cognition, 6(1), p.30. DOI: https://doi.org/10.5334/joc.285

## Correction

Due to a clerical error, the funding information in James et al. (2023) was incorrectly reported. DGW was supported on NIH grants R01 DC008774, R01 HD098652, and by the James S. McDonnell Foundation.
